# Exploring the effectiveness and experiences of people living with dementia interacting with digital interventions: A mixed methods systematic review

**DOI:** 10.1177/14713012241302371

**Published:** 2024-11-27

**Authors:** Annabel Ditton, Hissah Alodan, Christopher Fox, Shirley Evans, Jane Cross

**Affiliations:** 3286 University of Exeter, UK; 6106 University of East Anglia, UK; 3286 University of Exeter, UK; 8709 University of Worcester, UK; 6106 University of East Anglia, UK

**Keywords:** dementia, mild cognitive impairment, technology, quality of life, mixed methods

## Abstract

**Background:** As dementia care evolves, digital interventions are being developed to improve the quality of life of people living with dementia. It is also increasingly recognised that some people living with dementia can use and benefit from using digital interventions themselves. Therefore, exploring the effectiveness and experiences of using such interventions is essential to optimise digital intervention development and delivery.

**Method:** 5 databases were searched (MEDLINE (Ovid), PsycINFO, EMBASE, CINAHL and Web of Science) for papers reporting effectiveness outcomes or experiences, involving people living with dementia or mild cognitive impairment engaging with digital interventions for improving their quality of life. 73 relevant papers published between 2018–2023 were identified, 59 included effectiveness data and 18 included data on experiences.

**Results:** The integration of evidence identified that people living with dementia can benefit from engaging in digital interventions, if they are motivated, and provided with tailored training, support, appropriate devices and content. Benefits were seen within the domains of cognition, health and well-being and social relationships. Benefits were more frequent when digital interventions were provided in the home environment with specified daily/weekly usage requirements.

**Conclusion:** This review provides an overview of the current state of research exploring engagement of digital interventions by people with dementia for improving their quality of life. The findings provide guidance on how to optimise the method of delivery. Future research should explore how digital interventions can improve social relationships and self-concept of people living with dementia, the long-term sustainability of digital interventions, and how individuals with dementia form attitudes towards technology.

## Introduction

Over 55 million people worldwide have dementia (WHO, 2023). An additional 19.7% are living with mild cognitive impairment (MCI), which can develop into clinical dementia (Bruscoli et al., 2004; Busse et al., 2006; Song et al., 2023). The economic consequences total a global cost of US$23,796 per person with dementia, half of which is subsidised by informal care (Wimo et al., 2023). The personal consequences are challenging. Psychologically, people with dementia can experience grief, sadness, and loss of self-esteem (Aminzadeh et al., 2007; Lee et al., 2014; Scott, 2022). Functionally, changes can result in greater dependency on others and reduced relationship quality across social networks, and this can negatively impact on quality of life (Jing et al., 2016; Moyle et al., 2011; Nakayama et al., 2017; Victor et al., 2021). Thus, maintaining and improving the quality of life of people living with dementia and MCI is a research priority (Department of Health, 2015; Parkin & Baker, 2021).

Digital interventions, which can be defined as ‘Interventions delivered via information and communication technologies’, might improve quality of life and be cost effective (Murray et al., 2016). Newer forms recognise the active role individuals with dementia can play in using technology, leading to more interactive interventions such as cognitive stimulation or training, reminiscence opportunities, and virtual reality (Cipriani et al., 2006; Hayhurst, 2018; Irazoki et al., 2020; Lazar et al., 2014). There is also significant investment in digital interventions for people living with dementia to diagnose, monitor and intervene (Long et al., 2023). The effectiveness of assistive technology devices and telecare to achieve this is well documented (Chien et al., 2011; Pappada et al., 2021; Van der Roest et al., 2017). Reviews have also explored the effectiveness of digital interventions for improving outcomes such as social interaction, and wellbeing across multiple settings (Anderson et al., 2022; Bradley et al., 2023). Understanding the effectiveness of digital interventions that require engagement from people with dementia has also begun to be explored (Holthe et al., 2018), however, so far, these have not focused on whether interventions improve quality of life.

Dequanter et al. (2021), reviewed the development and effectiveness of digital interventions to support the quality of life of people living with dementia between 2013–18. In their review, they map interventions on to van Bronswijk, Bouma, and Fozard (2002) technology application domains. This framework focuses on how technology can be applied to improve older adult’s quality of life. Whilst their findings provide promising evidence that digital interventions can improve the quality of life of people with dementia and their carers, it is useful to explore digital interventions using a dementia specific quality of life framework.

Also, Dequanter et al.’s (2021) review does not consider how people living with dementia experience using interventions. Popular frameworks for developing complex interventions emphasise that development should focus on effectiveness, context, and implementation, to understand for whom, how and in what context the intervention may be useful (Skivington et al., 2021). To achieve this, exploring experience as well as effectiveness is essential.

This review aimed to fill this gap by answering the questions:

• What is the effectiveness of digital interventions which require interaction from people with dementia or MCI for improving their quality of life?
• What are the experiences of people with dementia or MCI when using these digital interventions?

Findings from this review will be used to develop understandings of what domains of quality of life can be improved by digital interventions and how intervention delivery might be optimised.

## Method

The reporting of this systematic review was guided by the standards of the Preferred Reporting Items for Systematic Review and Meta-Analysis (PRISMA) Statement (Moher et al., 2009). The protocol was registered on PROSPERO (CRD42023388030) in February 2023.

### Search strategy

Databases, MEDLINE [Ovid], PsycINFO, EMBASE, CINAHL and Web of Science were searched in February 2023 using MeSH terms and free text (supplemental material 1). Searches were supplemented by searches in Google Scholar and hand searching of reference lists. Additionally, RSS alerts were set for the specified search terms.

### Eligibility criteria

Inclusion and exclusion criteria were defined using the PEO framework (Capili et al., 2020).

#### Types of studies

Quantitative, qualitative and mixed-methods peer reviewed literature, written in English, and published between January 2018 - February 2023 were included.

#### Types of participants

Samples where at least 50% of participants were community dwelling people diagnosed with dementia or MCI, or samples containing dementia carer dyads were included. These were only included if participants had been diagnosed with MCI or dementia using a validated criterion. Validated criteria included diagnoses according to the Petersen (2001) criteria, the ATN (Clifford et al., 2016), NINCDS-ADRDA (McKhann et al., 1984), or diagnoses made by healthcare professionals. Community dwelling was defined as individuals living at home or in someone else’s home, including sheltered or support accommodation. We excluded people living in nursing homes, residential facilities, acute or psychiatric hospitals, and those receiving end of life care.

#### Types of interventions

Digital interventions, defined as ‘interventions delivered via information and communication technologies’ (Murray et al., 2016) were included if the participant had interacted with them. Interaction was defined as the person with dementia taking some control over the use of the intervention, independently or with support. This meant participants were required to use the intervention beyond the set-up period, such as pressing buttons, speaking into the device, or using their body to communicate and interact with the technology in some manner.

#### Types of outcomes

Quantitative data measuring any quality-of-life domain using a validated scale, as either a primary or secondary outcome were included. Outcomes were grouped according to the domains of quality of life defined by Smith et al.’s., (2005) conceptual framework of health-related quality of life. This framework is a dementia specific quality of life framework developed with people living with dementia and includes five concepts: daily activities and looking after yourself, health and wellbeing, cognitive functioning, social relationships, and self-concept. Additionally, qualitative data related to participants' experiences with digital interventions were included.

#### Study selection

Duplicates were removed using EndNote and RAYYAN software packages. Titles and abstracts were screened independently by two authors (AD and HA) for eligibility. Full versions of potentially relevant articles were obtained and reviewed for eligibility independently by AD and HA. Discrepancies were discussed, and if unresolved considered by the full research team for a decision.

### Data extraction

Data were extracted by one author (AD), and a second author (HA) independently extracted data from 7% of the articles to verify the accuracy of the extraction. There were no discrepancies, so remaining data were extracted by AD into bespoke tables based on the ‘Cochrane Collaboration Data Collection Tools’ (Li et al., 2023). Extracted data included:

• Participants: age, gender, diagnosis, ethnicity, socio-demographic background, sample size, baseline cognitive status.
• Methods: study location, publication date, study design, recruitment strategy, attrition, follow up, setting.
• Interventions: intervention name, software, hardware, support given to use intervention, length and frequency of intervention, control group information.
• Outcomes: primary and secondary quantitative outcomes, direct participant quotes, author interpretations, observations, reflexive notes, and thematic analysis themes.

### Quality assessment

Study quality was assessed using appropriate Joanna Briggs Institute (JBI) quality assessment checklists. The Mixed Methods Appraisal Tool (MMAT) was used for mixed methods studies (Hong et al., 2018).

Article quality was graded by author AD with 7% also being independently graded by HA for accuracy checking. Checklist items were graded as ‘yes’, ‘no’, ‘can’t tell’, and ‘not applicable’. Discrepancies were minimal (12%). All articles were then re-evaluated for quality by AD based on HA’s comments with AD making the final decision.

Each article received a quality percentage score based on the number of checklist criteria they met using the formula $\left(Item\;graded\;'yes'÷All\;items\right)\times 100$ . Items graded ‘not applicable’ were not included in calculations. Quality was then narratively labelled as high (≥70%), moderate (51%–69%) and low (≤50%) but no articles were removed based on their quality score.

Traditionally in qualitative meta-syntheses, credibility of individual findings is graded based on the number of supporting illustrations using CONQUAL (Munn, 2014a). This categorises study findings as either supported or unsupported. Unsupported findings are usually excluded from analyses. The complexities of data integration means that mixed-method review guidance does not recommend grading and excluding qualitative findings based on credibility (Stern et al., 2020). Therefore, all findings from the qualitative articles are included, even if not supported by illustrations.

### Methods of data synthesis

A convergent segregated approach was used to synthesise data in accordance with JBI guidance and methodological papers (Heyvaert et al., 2011; Hong et al., 2017; Stern et al., 2020). This involved synthesising quantitative and qualitative data separately and integrating them at the end to form a narrative.

Quantitative data was narratively synthesised to determine effectiveness (Campbell et al., 2020). Articles were grouped by quality-of-life domain, determined by the outcome measures used. Several articles measured multiple quality of life domains and are therefore repeated within the synthesis. Vote counting based on direction of effects with sign test statistics were used to determine intervention effects using mean difference values, effect sizes and p statistics. This involved categorising each outcome as showing benefit or harm, regardless of effect size or statistical significance, creating a binary metric (McKenzie et al., 2023). The metrics were then grouped by quality-of-life domain and a sign test statistic was used to determine the presence of a positive effect (McKenzie et al., 2023). Sign test statistics that reached significance (p = ≤0.05) demonstrated that digital interventions positively influenced quality of life.

Qualitative data was synthesised using meta-aggregation (Lockwood et al., 2015). This involved extracting all findings produced by authors of the included articles, grouping these into categories, and grouping categories to form synthesised review findings. The aim of producing synthesised findings was to provide recommendations for optimising intervention delivery (Munn, Tufanaru, & Aromataris, 2014).

Both syntheses were then integrated together by juxtaposing and linking the findings to produce a third synthesis (Harden, 2010; Stern et al., 2020). This synthesis is presented in narrative recommendations on how digital interventions can benefit the quality of life of people living with dementia, and how delivery may be optimised.

## Results

### Included studies

**Figure 1.**
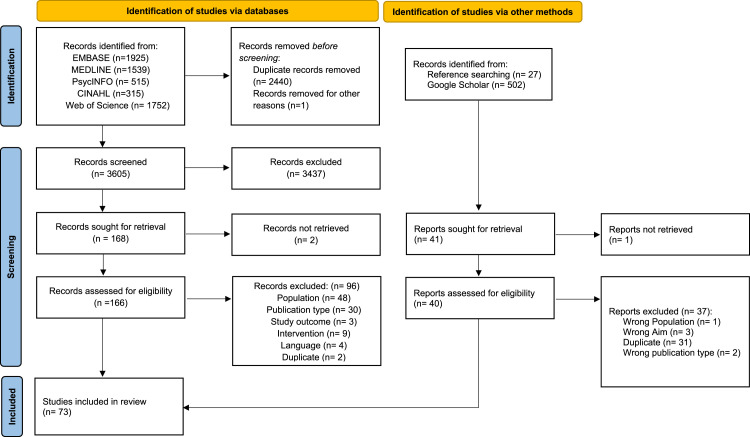
PRISMA flowchart for study selection.

6046 citations were retrieved from electronic databases and an additional 529 citations were retrieved from additional searches, resulting in a total of 6,575 publications. Of these, 73 articles met eligibility. 59 contained quantitative data related to quality-of-life outcomes, and 18 contained qualitative data related to experience (Figure 1) (Page et al., 2021). Study characteristics for all studies can be found in Table 1.

**Table 1.** Study characteristics of included studies.

Study	Country	Design	Sample (n)	Intervention duration	Digital intervention	Method of delivery	Quality of life domains explored	Outcome measures
Beentjes, Neal, et al. (2020)	Netherlands	RCT	PwD/MCI (47)	3 months	FindMyApps	Self-guided at home	Social relationships, self-concept, quality of life	ASCOT, MSPP, self-management ability scale – Short version, Dutch general self-efficacy scale, DQoL
Chen et al. (2022)	Taiwan	Non-randomised	VD (98)	10 weeks	Computerised cognitive remediation therapy	n/r	ADL, social relationships, cognition, quality of life	Activities of daily living, personal and social performance scale, MMSE, GQOL-74
Cheng et al. (2022)	n/r	RCT	MCI + Parkinson’s (40)	3 months	Virtual reality cognitive training	n/r	Health and wellbeing, cognition	BDI, RBANDS, MOCA
Cinar and Sahiner (2020)	Turkey	Non-randomised	Probable AD (60) SCI (60)	3 months	Beynex: Web-based exergame	Self-guided at home	ADL, health and wellbeing, cognition	Bayer activities of daily living scale, GDS, MOCA, Cambridge cognition CANTAB assessment
Diaz Baquero et al. (2022)	Spain, Netherlands	RCT	MCI/PwD (62)	12 months	GRADIOR: Computerised cognitive training	Supported at day centre	Health and wellbeing, cognition	GDS, Alzheimer’s disease assessment scale: Cognitive subscale, CAMCOG, Clock Drawing test, Lexical verbal Fluency, MMSE, RBMT, Semantic verbal Fluency, TMT
Duff et al. (2022)	United States	RCT	MCI (113)	1 year	BrainHQ©: Web based cognitive training	Self-guided at home	ADL, cognition	Alzheimer disease Cooperative study-activities of daily living, RBANS
Edgar and Bargmann (2021)	United States	Case study	AD/VD (2)	2 years, 4	Constant Therapy®: Cognitive training application	Supported at clinic or via teleconference	Cognition	MOCA, Arizona Battery for communication Disorders of dementia, cognitive Linguistic Quick test
Fasili et al. (2018)	Greece	Case series	PwD (10)	7 weeks	Virtual reality cognitive training	Supported at clinic	Cognition	Digit span test, Babcock story recall, TMT, Hanoi Tower, Wisconsin Card Sorting Test-64, executive functions (FAB)
Ferry et al. (2020)	United Kingdom	Cost effectiveness analysis	PwD (30)	12 weeks	Reminiscence application	Self-guided at home	Quality of life	DEMQOL, DEMQOL Proxy, euro-QoL-5D
Flak et al. (2019)	n/r	RCT	MCI (68)	4 months	CogMed®: Computerised cognitive training	Self-guided at home	Cognition	Wechsler memory Scale-3, Delis–Kaplan executive function system, California verbal learning Test-2, RCFT
Foloppe et al. (2018)	France	Case study	Probable AD (1)	6 months	Virtual reality ADL training	Supported at home	ADL, cognition, self-concept, quality of life	Instrumental activities of daily living scale, MMSE, rosenburg self-esteem scale, AD-QoL
Han et al. (2020)	Korea	RCT	MCI (24)	12 weeks	Computerised cognitive training	Supported in group environment	Health and wellbeing, cognition, quality of life	BDI, MMSE, Korean-MOCA, quality of life- Alzheimer disease
Imbeault, Gagnon, et al. (2018)	n/r	Case study	AD (3)	19 months, 1 year, 2 years	Assistive technology application	Self-guided at home	Cognition, health and wellbeing	Prospective and retrospective memory questionnaire, Multifactorial memory Questionnaire, GDS
Imbeault, Langlois, et al. (2018)	n/r	Case study	Probable AD (1)	1 year	Assistive technology application	Self-guided at home	Cognition, health and wellbeing	Prospective and retrospective memory questionnaire, Multifactorial memory Questionnaire, GDS
Jirayucharoensak et al. (2019)	Thailand	RCT	MCI (65) Healthy adults (54)	3 months	Exergame with neurofeedback	Supported at clinic	Cognition	Cambridge neuropsychological test automated Battery, MOCA, Thai-MMSE, delayed Matching to Sample test, Pattern recognition memory test, rapid Visual information processing
Kerkof et al. (2022)	n/r	Mixed methods	MCI (11)	3 months	FindMyApps	Self-guided at home with telephone support	ADL, health and wellbeing, social relationships, self-concept, quality of life	Experienced autonomy list, Pleasant activities list, ASCOT, self-management ability scale, Dutch general self-efficacy scale, DQoL
Kim et al. (2021)	Korea	Non-randomised	MCI (22) healthy adults (22)	4 weeks	Virtual reality cognitive training	Observed by researcher	Cognition	CERAD, cognitive reserve Index
Kim et al. (2020)	Korea	RCT	MCI (32)	20 weeks	RehaCom: Computerised cognitive training	n/r	ADL, health and wellbeing, cognition, quality of life	Instrumental activities of daily living scale, activities of daily living, centre for epidemiological studies depression scale, Alzheimer’s disease assessment scale, MOCA, euro-QOL-5D
Knoefel et al. (2018)	n/r	RCT	MCI (16)	9 weeks	BrainHQ©: Web based cognitive training	n/r	Cognition	MOCA, TMT, RBANS
König et al. (2022)	n/r	Mixed methods	MCI/AD (30)	12 weeks	Memento: Web-based exergame	Self-guided at home with telephone support	ADL, health and wellbeing, cognition, quality of life	Alzheimer disease Cooperative study-activities of daily living, NPI, world health Organization Disability assessment schedule, QOL-AD
Laird et al. (2018)	United Kingdom	Non-randomised	PwD (30)	19 weeks	InspireD©: Reminisce application	Self-guided at home	Health and wellbeing, social relationships	WHO-5, mutuality scale, the quality of carer patient relationship scale
Lee et al. (2018)	Korea	RCT	MCI/AD/VD (20)	3 weeks	Comcog: Computerised cognitive training, Bettercog: Computerised cognitive training	Observed at clinic	ADL, cognition	Korean version modified Barthel index, Korean-MMSE, CDR, Seoul neuropsychological screening Battery
Li et al. (2019)	China	RCT	MCI/AD (141)	18 months	Web-based cognitive training	Self-guided at home	Cognition	MMSE, ACE-R, auditory verbal learning test, Shape Trail test, SDST, Stroop colour test
Maeng et al. (2021)	Korea	Non-randomised	MCI (24) healthy adults (23)	4 weeks	Virtual reality based cognitive therapy	Observed by clinician	Cognition, quality of life	DGS, Korean- CERAD, Korean-QOL-AD
Manenti et al. (2020)	n/r	RCT	MCI (49)	7 months	Virtual reality based cognitive therapy	Observed by clinician	Cognition, quality of life	Rey auditory verbal learning test, free and Cure selective reminding test, verbal fluency phoemic, verbal fluency semantic, Battery for analysis of aphasic deficits, TMT, Clock Drawing test, QOL-AD
Marin et al. (2022)	United States	RCT	MCI/AD (19)	42 weeks	Constant Therapy®: Cognitive training application	Self-guided at home	Cognition	RBANS
Mattos et al. (2021)	n/r	Mixed methods	MCI (10)	10 weeks	CBT-i: Web-based sleep intervention	Self-guided at home	Cognition	MOCA, Blind-MOCA
Micarelli et al. (2019)	n/r	RCT	MCI (24) healthy adults (23)	4 weeks	Exergame	Self-guided at home	Health and wellbeing	Dizziness handicap Inventory (emotional, physical, functional), Dynamic Gait Index, activities-specific balance confidence scale
Nousia et al. (2021)	Greece	RCT	MCI (46)	15 weeks	RehaCom: Computerised cognitive training	n/r	Cognition	MOCA, Clock Drawing test, immediate word recall, word recognition test and delayed word memory test, Boston naming test, Semantic Fluency, Digit span test, TMT
Park, Jung, and Lee (2020)	South Korea	RCT	MCI (35)	6 weeks	MOTORCOG®: Virtual reality exergame	Supported by clinician	Cognition	MOCA, TMT, Digit span test
Park, Yun, et al. (2020)	South Korea	RCT	MCI (21)	6 weeks	Virtual reality cognitive training	Observed by researcher	Cognition	Korea-CERAD, Korean-MMSE, CDR
Park, Liao, et al. (2020)	South Korea	RCT	MCI (21)	3 months	Virtual reality cognitive training	Supported by clinician at clinic	Cognition, health and wellbeing	Korean-GDS, Korean-MMSE, Digit span test, the Stroop test
Park (2022a)	South Korea	RCT	MCI (56)	8 weeks	Virtual reality cognitive training	Supported by clinician	Cognition	WAIS, Shiraz verbal learning test
Park (2022b)	South Korea	RCT	MCI (32)	8 weeks	Virtual reality cognitive training	Supported by clinician	Cognition, ADL	Korean instrumental activities of daily living, executive function performance test
Petersen et al. (2020)	Denmark	Non-randomised	PwD (23)	27 weeks	Web-based exercises	Supported by clinician at clinic followed by self-guided at home	Health and wellbeing, cognition, quality of life	Sit to Stand, Timed-up-and-go (s), 10-meter dual walking task (s), 6-minute walking tst (meters), NPI, MMSE, euro-QOL-5D
Phatak et al. (2021)	n/r	Cluster-RCT	MCI (272)	12 months	BrainHQ©: Web based cognitive training	Self-guided at home	Cognition	Cogstate brief Battery
Poptsi et al. (2019)	Greece	RCT	MCI (71)	6 months	Computerised cognitive training	Supported in group environment	ADL, cognition	Functional rating scale for symptoms of dementia, functional cognitive assessment, MMSE, rey auditory verbal learning test, RCFT, rivermead behavioural assessment, verbal fluency test, Pyramids and Palms, Boston Aphasia examination
Rai, Schneider, and Orrell (2021)	United Kingdom	RCT	PwD (61)	11 weeks	iCST: Cognitive stimulation application	Self-guided at home	ADL, health and wellbeing, social relationships, cognition, quality of life	Bristol activities of daily living scale, Cornell scale for depression in dementia, NPI, quality of carer patient relationship Questionnaire, Alzheimer’s disease assessment scale-cognition, QOL-AD, euro-QOL-AD
Rodella et al. (2022)	Italy	RCT	MCI/AD (28)	6 months	CoRe: Computerised cognitive training	Supported in laboratory	Health and wellbeing, cognition, quality of life	BDI, MMSE, Logical memory test, Rey’s 15 words test, RCFT, raven Matrices, Frontal assessment Battery, Semantic and Phonological fluencies, Digit span test, Corsi’s Block Tapping test, Attentive Matrices, Trails making test, short form 36 health Survey Questionnaire
Sautter et al. (2021)	United States	RCT	AD (18)	6 weeks	iN2L: Multi-functional computer	Self-guided at home	Health and wellbeing, cognition	Affect balance scale, GDS, MOCA
Schmitter-Edgecombe et al. (2021)	n/r	RCT	MCI (29)	5 months	Assistive technology application	Self-guided at home	ADL, cognition, self-concept, quality of life	Instrumental activities of daily living scale, RBANS, Coping self-efficacy scale, QOL-AD, Satisfaction with life scale
Scullin et al. (2022)	n/r	RCT	MCI/PwD (48)	4 weeks	Assistive technology application	Self-guided at home	ADL, cognition, quality of life	Instrumental activities of daily living scale, Prospective, retrospective memory Questionnaire, Neuro-QoL
Sheehy et al. (2022)	Canada	Mixed methods	MCI (11)	6 weeks	Virtual reality games	Self-guided at home	Health and wellbeing, cognition, social relationships	Berg balance scale, community balance and Mobility scale, timed up and go test, functional lower-extremity strength test, community integration Questionnaire, RBANS
Shyu et al. (2022)	Taiwan	RCT	AD/VD/DwL (16)	3 months	Computerised cognitive training	Supported by nurses at home	Health and wellbeing, cognition, quality of life	GDS, cognitive abilities screening Instrument, MMSE, PRMQ, CDR, Chinese-DQoL
Singh et al. (2022)	n/r	RCT	MCI (49)	Not reported	Virtual reality mindfulness	n/r	Health and wellbeing, cognition	Mindfulness attention awareness scale, Loewenstein occupational therapy cognitive assessment-Geriatric
Taylor et al. (2020)	n/r	Non-randomised	PwD (15)	12 weeks	Exercise application	Self-guided at home	Health and wellbeing, cognition	GDS, short physical performance Battery, timed up and go, MOCA, TMT, Falls efficacy scale
Thapa et al. (2020)	South Korea	RCT	MCI (66)	8 weeks	Virtual reality cognitive training	Supported by clinician	Health and wellbeing, cognition	Gait speed test, 8 ft up and go test, hand-grip strength, MMSE, national Center for Geriatrics and Gerontology functional assessment tool, TMT, SDST
Tsolaki et al. (2020)	Greece	Non-randomised	MCI (244)	12 weeks	BrainHQ©: Web based cognitive training	Supported in group environment	ADL, health and wellbeing, cognition	Functional and cognitive assessment test, functional rating scale of symptoms of dementia, instrumental activities of daily living scale, GDS, Beck Anxiety Inventory, BDI, MMSE, MOCA, rey-auditory verbal learning test, California verbal learning test, TMT, Digit span test
Yang et al. (2022)	Korea	RCT	MCI (99)	8 weeks	Virtual reality cognitive training	n/r	Health and wellbeing, cognition	Gait speed test, hand grip strength, Appendicular skeletal muscle mass, MMSE, TMT, SDST
Zajac-Lamparska et al. (2019)	n/r	Non-randomised	PwD (27)	4 weeks	GRADYS: Virtual reality cognitive training	Observed by researcher	Cognition	WAIS, Colour Trail test, D2 test of attention, Digit span test, Benton Visual retention test, the Famous Faces test, RCFT, ACE-III, Boston Naming test
Zhu et al. (2022)	China	Non-randomised	MCI (31)	5 weeks	Virtual reality cognitive training	Supported by clinician at care centre	Health and wellbeing, cognition	GDS, Perceived stress scale, MMSE, MOCA Symbol Digit Modality test, auditory verbal learning test, Shape Trail test
van Stanten et al. (2020)	n/r	Cluster-RCT	PwD (84)	6 months	Web-based exergame	Supported at day centre	Health and wellbeing, cognition Social relationships, quality of life	Physical activity scale of the elderly, euro-QOL-5D, ASCOT, MMSE, TMT
Bojan et al. (2021)	Greece	Non-randomised	MCI/AD/VD (30)	1 day	Cosma: Multi-functional application	Supported in laboratory	Health and wellbeing	Positive and negative affect scale
Amjad et al. (2019)	Pakistan	RCT	MCI (38)	6 weeks	Dr. Kawashimsa: Virtual reality cognitive training	Observed at clinic	Cognition	MMSE, MOCA, TMT
Torpil et al. (2021)	n/r	RCT	MCI (68)	12 weeks	Virtual reality cognitive training	Observed at clinic	Cognition	Loewenstein occupational therapy cognitive assessment-Geriatric
Burgos-Morelos et al. (2023)	n/r	Non-randomised	MCI/PwD (38)	3 weeks	Computerised cognitive training	n/r	Health and wellbeing	Grooved Peg board test, Minnesota manual dexterity test
Cavallo et al. (2019)	n/r	RCT	Probable AD (36)	1 year	Brainer©: Computerised cognitive training	n/r	Cognition	MMSE, Digit span test, RBMT, graded naming test, Visual Object and Space perception Battery, verbal Fluency test, Hayling & Brixton test
Debring et al. (2021)	Sweeden	Non-randomised	PwD (118)	12 weeks	Circa: Web-based communication aid, CIRCA: Web-based communication aid	Self-guided at home	Cognition	Euro-QOL-5D, QOL-AD
Zhang et al. (2019)	China	Non-randomised	MCI (17)	12 weeks	Computerised cognitive training	Observed at senior centre	Cognition	MMSE, MOCA, Hopkins verbal learning test, TMT, brief Visuospatial memory test, verbal Fluency test, Digit span test, Stroop-word test, Stroop-colour test, Folden’s interference score, Modified picture completion test
Beentjes, Kerkhof, et al. (2020)	Netherlands	Mixed-methods	PwD/MCI (20)	3 months	FindMyApps	Self-guided at home		
Beishon et al. (2021)	n/r	Mixed-methods	MCI (6), AD (12), healthy adults (10)	13 weeks	Lumosity: Cognitive training application	Self-guided at home		
Berenhaum et al. (2020)	Israel	Mixed-methods	PwD (19)	1 day	“My brain works”: Cognitive training games	Supported by researcher		
Berrett et al. (2022)	Australia	Qualitative	PwD (28)	18 months	Assistive technologies	Supported by researcher at home		
Bielsten et al. (2020)	Sweeden	Qualitative	PwD (6)	n/r	DemPower: Reminiscence application	Self-guided at home		
Bogza et al. (2020)	n/r	Mixed methods	MCI (12)	1 day	Web-based decision aid	Supported by researcher		
Critten and Kucirkova (2019)	n/r	Qualitative	PwD (3)	n/r	OurStory: Reminiscence application	Self-guided at home		
Flynn et al. (2022)	n/r	Qualitative	PwD (9)	1 day	FOUNDations: Virtual reality games	Supported by researcher as a one-off trial		
Hicks et al. (2019)	United Kingdom	Qualitative	PwD (16)	9 weeks	Various multi-functional devices and applications	Supported in group environment		
Øksnebjerg et al. (2019)	n/r	Qualitative	Probable AD/PwD (11)	10 weeks	Various multi-functional devices and applications	Self-guided at home		
Rai, Griffiths, et al. (2021)	United Kingdom	Mixed methods	PwD (13)	1 day	iCST: Cognitive stimulation application	Observed during interview		
Rai, Prasetya, et al. (2021)	Indonesia	Mixed methods	PwD (6), caregivers (12)	1 day	iCST: Cognitive stimulation application	Observed during interview		
Ryan et al. (2020)	United Kingdom	Qualitative	PwD (15), caregivers (17)	12 weeks	InspireD©: Reminisce application	Self-guided at home		
Smith et al. (2020)	United Kingdom	Qualitative	PwD (12)	1 day	Various multi-functional devices and applications	Supported in group environment		

Abbreviated terms: PwD, Person with Dementia type unspecified; ADL; Daily activities and looking after yourself; ASCOT, Adult Social Care Outcomes Toolkit; MSPP, Maastricht Social Participation Profile; DQoL, Dementia Quality of Life Instrument; MMSE, Mini-Mental State Examination; GQOL-74, Generic Quality of Life Inventory-74; BDI, Becks Depression Inventory; RBANS, Repeatable Battery for the Assessment of Neuropsychological Status; MOCA, Montreal Cognitive Assessments; GDS, Geriatric Depression Scale; CAMCOG, Cambridge Cognition Examination; RBMT, Rivermead Behaviour Memory Test; TMT, Trails Making Test; DQOL, Dementia Quality of Life Measure; Euro-QoL-5D, European Quality of Life Five Dimension Five Level Scale; RCFT, Rey Complex Figure Test; AD-QoL, Alzheimer’s Disease-Related Quality of Life; DQOL, Dementia Quality of Life Instrument; CERAD, Consortium to Establish a Registry for Alzheimer’s Disease; NPI, Neuropsychiatric Inventory; QOL-AD, Quality of Life- Alzheimer Disease Scale; CDR, Clinical Dementia Rating; ACE-R, Addenbrookes Cognitive Examination-Revised; ACE-III, Addenbrookes Cognitive Examination; SDST, Symbol Digit substitution Test; WAIS, Wechsler Adult Intelligence Scale; PRMQ, Prospective and retrospective Memory Questionnaire.

### Quality of articles

Overall study quality varied. RCT’s were mostly of moderate quality (Park, 2022a; Park, Liao, et al., 2020; Park, Jung, & Lee, 2020; Poptsi et al., 2019; Schmitter-Edgecombe, 2022; Scullin et al., 2022; Shyu et al., 2022; Thapa et al., 2020; Torpil et al., 2021), twelve were of low quality (Amjad et al., 2019; Beentjes, Neal et al., 2020; Han et al., 2020; Jirayucharoensak et al., 2019; Knoefel et al., 2018; Li et al., 2019; Marin et al., 2022; Park 2020a; Phatak et al., 2021; Sautter et al., 2021; Singh et al., 2022; Yang et al., 2022). They commonly failed to provide sufficient detail of concealment, blinding, details of power calculations, or justifications of sample size. Non-randomised studies were mostly moderate quality (Bojan et al., 2021; Debring et al., 2021; Kim et al., 2021; Maeng et al., 2021; Petersen et al., 2020; Tsolaki et al., 2020; Zhang et al., 2019; Zhu et al., 2022), two were low quality (Burgos-Morelos et al., 2023; Chen et al., 2022). Commonly these lacked control groups, incomplete outcomes, lacked detail regarding the reliability of outcome measurement, showed no power calculations, or justifications for sample sizes. Mixed Methods designs were all high quality. Missing was a lack of description on the impact of confounding variables and descriptions related to risk of non-response biases. Case study designs were high quality, except for the case series design which was low in quality (Fasilis et al., 2018). This lacked clear descriptions of the participant information and reporting of site demographics. All qualitative studies were high quality except Øksnebjerg et al. (2019) which was low quality. This study failed to describe philosophical and methodological perspectives, had unclear analytical methods, and provided insufficient participant data to determine adequate representation of participants. They also did not locate the researcher theoretically or culturally in the study, a common flaw amongst 55% of the qualitative studies (Berrett et al., 2022; Critten & Kucirkova, 2019; Ryan et al., 2020; Øksnebjerg et al., 2019). The quality assessment checklist scores for all studies are presented in supplemental material 2.

### Quantitative Synthesis

#### Study characteristics

Most articles were randomised control trials (*n* = 35, see Table 1). Study designs also included non-randomised control trials (Cinar & Sahiner, 2020; Kim et al., 2021; Maeng et al., 2021; Petersen et al., 2020; Tsolaki et al., 2020), within subject designs (Bojan et al., 2021; Burgos-Morelos et al., 2023; Chen et al., 2022; Debring et al., 2021; Laird et al., 2018; Taylor et al., 2020; Zajac-Lampaska et al., 2019; Zhang et al., 2019; Zhu et al., 2022), mixed methods (Kerkhof et al., 2022; König et al., 2022; Mattos et al., 2021; Sheehy et al., 2022), case studies (Edgar & Bargmann, 2021; Foloppe et al., 2018; Imbeault et al., 2018a, 2018b), case series (Fasilis et al., 2018), and a cost effectiveness design (Ferry et al., 2020). Twenty articles did not report their location. Of those that did, locations included Europe (Diaz Baquero et al., 2022; Beentjes, Neal, et al., 2020; Bojan et al., 2021; Debring et al., 2021; Fasilis et al., 2018; Ferry et al., 2020; Foloppe et al., 2018; Laird et al., 2018; Marin et al., 2022; Nousia et al., 2021; Petersen et al., 2020; Poptsi et al., 2019; Rai, Schneider, & Orrell, 2021; Rodella et al., 2022; Sautter et al., 2021; Tsolaki et al., 2020), Republic of Korea (Han et al., 2020; Kim et al., 2020, 2021; Lee et al., 2018; Maeng et al., 2021; Park, 2022a, 2022b; Park et al., 2020a, 2020b; Park, Jung, & Lee, 2020; Thapa et al., 2020; Yang et al., 2022), China (Li et al., 2019; Zhang et al., 2019; Zhu et al., 2022), United States of America (Duff et al., 2022; Edgar & Bargmann, 2021), Taiwan (Chen et al., 2022; Shyu et al., 2022), Pakistan (Amjad et al., 2019), Canada (Sheehy et al., 2022), Turkey (Cinar & Sihinar, 2020), and Thailand (Jirayucharoensak et al., 2019).

The duration of interventions ranged between <1 day to 4 years, with most providing interventions for 12 weeks (Beentjes, Neal, et al., 2020; Cavallo & Angilletta, 2019; Cinar & Sahiner, 2020; Debring et al., 2021; Duff et al., 2022; Ferry et al., 2020; Kerkhof et al., 2022; König et al., 2022; Laird et al., 2018; Manenti et al., 2020; Park, Liao, et al., 2020; Torpil et al., 2021; Tsolaki et al., 2020; Zhang et al., 2019). Most designs contained two groups such as digital intervention versus control, digital intervention versus non-digital intervention, or cognitively impaired versus healthy adults. Seven articles compared three or more different arms (Cheng et al., 2022; Cinar & Angilletta, 2019; Jirayucharoensak et al., 2019; Manenti et al., 2020; Poptsi et al., 2019; Tsolaki et al., 2020; Yang et al., 2022).

Sample sizes ranged from 1 to 272 participants, with a total 2793 participants providing quantitative data on quality-of-life outcomes. The diagnosis of participants consisted primarily of individuals with mild cognitive impairment (*n* = 1697). Other diagnoses included individuals with Alzheimer’s disease (*n* = 21), vascular dementia (*n* = 98), unspecified or combination of dementias (*N* = 416), mixed samples of mild cognitive impairment or dementia (*n* = 463), and probable Alzheimer’s disease (*n* = 98). Only 17 studies reported age range of participants which ranged from 50 to 101 years, with an average age of 74 years (Beentjes, Neal, et al., 2020; Chen et al., 2022; Debring et al., 2021; Edgar & Bargmann, 2021; Ferry et al., 2020; Flak et al., 2019; Foloppe et al., 2018; Imbeault et al., 2018a, 2018b; König et al., 2022; Laird et al., 2018; Marin et al., 2022; Mattos et al., 2021; Poptsi et al., 2019; Sheehy et al., 2022; Torpil et al., 2021; Zajac-Lamparska et al., 2019). Ethnicity was only reported in nine studies, however, both western and eastern ethnicities were represented (Edgar & Bargmann, 2021; Li et al., 2019; Marin et al., 2022; Nousia et al., 2021; Park, 2022a, 2022b; Rai, Schneider, & Orrell, 2021; Schmitter-Edgecombe et al., 2022; Scullin et al., 2022).

Digital interventions were categorised by their hardware and software. Virtual reality software was present in 21 interventions. These included cognitive training and stimulation (Amjad et al., 2019; Flak et al., 2019; Foloppe et al., 2018; Kim et al., 2021; Maeng et al., 2021; Manenti et al., 2020; Park, Yun, et al., 2020; Park, Liao, et al., 2020; Park, 2022a; Thapa et al., 2020; Torpil et al., 2021; Yang et al., 2022; Zajac-Lamparska et al., 2019; Zhu et al., 2022), leisure and relaxation (Fasilis et al., 2018; Flynn et al., 2022; Cheng et al., 2022; Sheehy et al., 2022; Singh et al., 2022), and for physical exergames (Micarelli et al., 2019; Park, Jung, & Lee, 2020). Computerised software was present in 45 studies which included web pages, applications, smart devices, and computer programs. These consisted of cognitive training or stimulation interventions (*n* = 23, see Table 1), interventions for improving activities of daily living (Beentjes, Kerkhof, et al., 2020; Beentjes, Neal, et al., 2020; Berret et al., 2022; Imbeault et al., 2018a, 2018b; Kerkhof et al., 2022; König et al., 2022; Schmitter-Edgecombe et al., 2022; Scullin et al., 2022; Smith et al., 2020; Øksnebjerg et al., 2019), physical exergames or exercises (Cinar & Sahiner, 2020; Jirayucharoensak et al., 2019; Petersen et al., 2020; Taylor et al., 2020; Tsolaki et al., 2020; van Stanten et al., 2020), reminiscence opportunities (Bielsten et al., 2020; Critten & Kucirkova, 2019; Ferry et al., 2020; Laird et al., 2018; Ryan et al., 2020), leisure activities (Berenbaum et al., 2020; Hicks et al., 2019), and a communication aid (Debring et al., 2021). Also present was a dementia specific computer (Sautter et al., 2021), and a website providing cognitive behavioural therapy for insomnia (Mattos et al., 2021). Several interventions were explored in multiple studies. These included FindMyApps, an application to assist people to download dementia friendly tablet apps (Beentjes, Kerkhof, et al., 2020; Beentjes, Neal, et al., 2020; Kerkhof et al., 2022), BrainHQ©, a computerised cognitive training website (Duff et al., 2022; Knoefel et al., 2018; Phatak et al., 2021; Tsolaki et al., 2020), iCST, an application for cognitive stimulation (Rai et al., 2021a, 2021b, 2021c), InspireD, a reminiscence application (Laird et al., 2018; Ryan et al., 2020), and Constant Therapy®, a computerised cognitive training website (Edgar & Bargmann, 2021; Marin et al., 2022).

**Figure 2.**
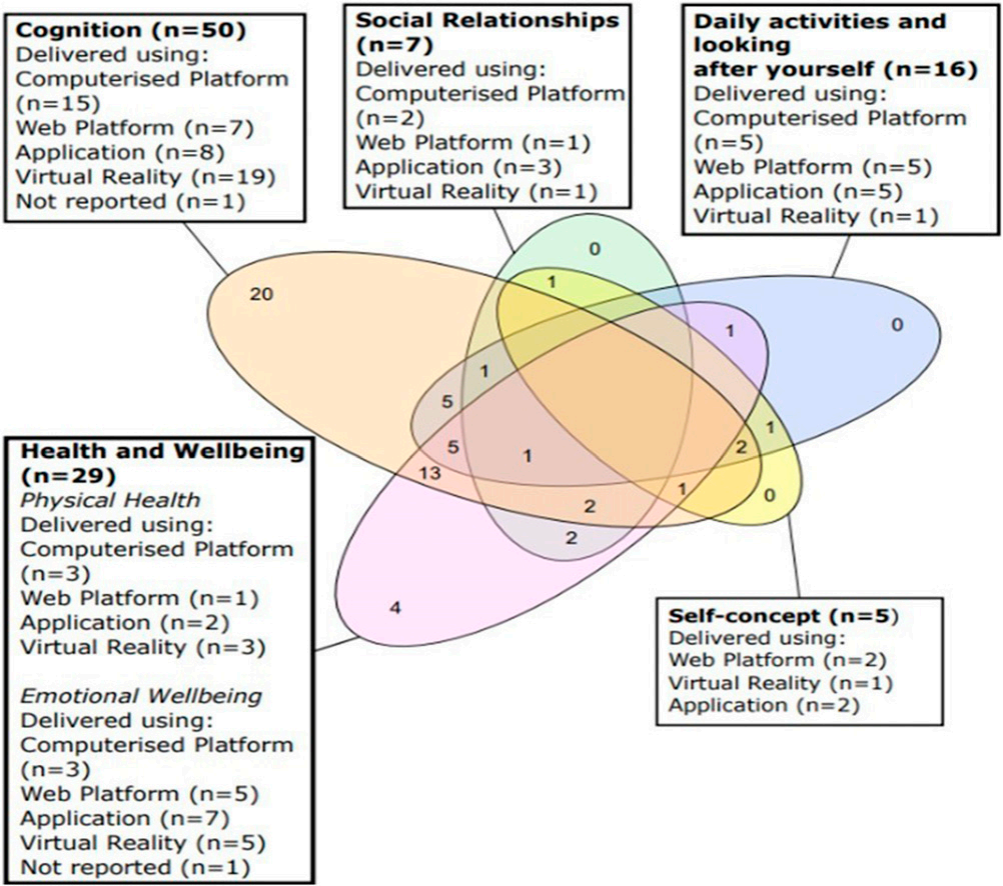
Venn diagram highlighting the combinations of quality-of-life domains measured in studies, and which digital platforms were used.

Seventeen articles explored quality of life as a whole measure additional to exploring individual quality-of-life domains (Beentjes, Neal, et al., 2020; Chen et al., 2022; Foloppe et al., 2018; Han et al., 2020; Kerkhof et al., 2022; Kim et al., 2021; König et al., 2022; Maeng et al., 2021; Manenti et al., 2020; Petersen et al., 2020; Rai, Schneider, & Orrell, 2021; Rodella et al., 2022; Schmitter-Edgecombe et al., 2022; Scullin et al., 2022; Shyu et al., 2022; van Stanten et al., 2020), two explored quality-of-life as a whole measure without exploring individual domains (Debring et al., 2021; Ferry et al., 2020). Figure 2 shows the combination of quality-of-life domains explored within articles, and the number of digital methods used by interventions to explore each domain. Cognition was the most explored domain with mostly virtual reality interventions. Twenty articles only explored cognition, thirteen explored cognition and health and wellbeing, five cognition and daily activities and looking after yourself, and five explored all three of these domains. Self-concept and social relationships were least explored, and outcomes related to these appeared in five and seven articles respectively. Measures used to explore quality of life varied (see Table 1). Tables 2 and 3 show the number of outcome measures used in each study and the direction of outcome effects for each quality-of-life domain per study.

**Table 2.** Direction of effects for randomised control trials.

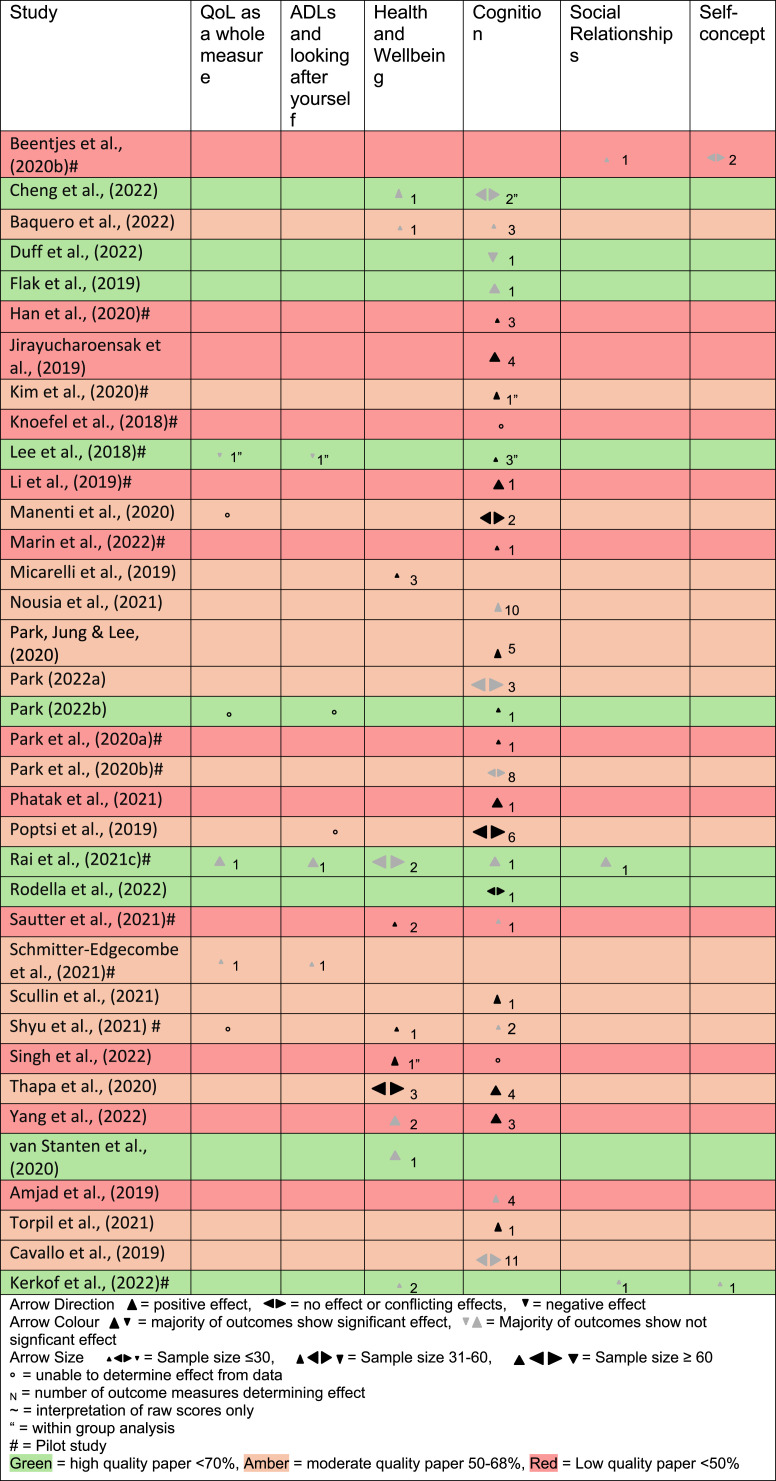

**Table 3.** Direction of effects for non-randomised trials.

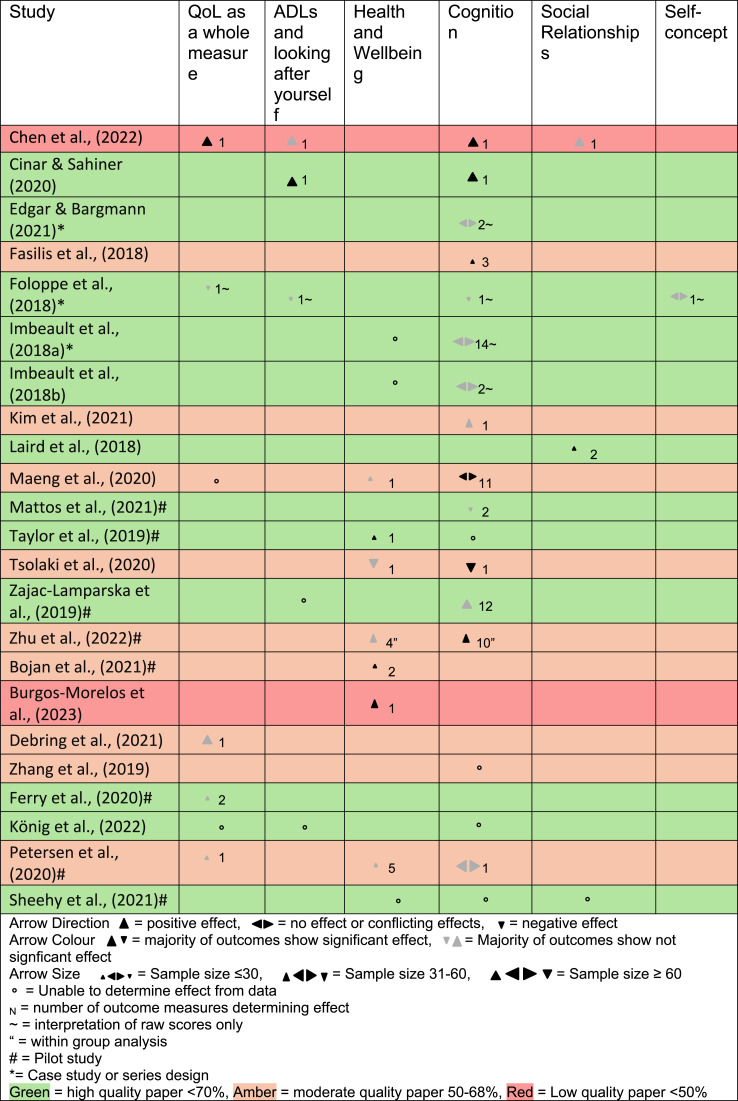

#### Effectiveness of digital interventions

Using means, standard deviations, effect sizes, and the narrative descriptions from authors, sign test statistics were used to detect the presence of an effect for digital interventions improving each quality-of-life domain (Table 4). These show digital interventions positively affect cognition, social relationships and health and wellbeing domains.

**Table 4.** Direction of effects with sign test statistics per quality-of-life domain.

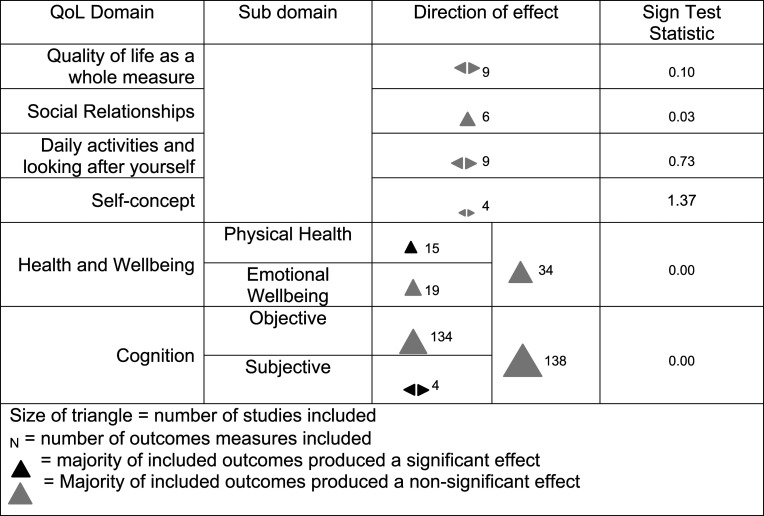

##### Cognition

Fifty articles explored cognition, data were included from 43 studies using cognition as the primary outcome or did not specify that it was not the primary outcome (see Tables 2 and 3). One hundred and thirty-eight outcome effects were identified across cognitive domains; however, most did not reach statistical significance. When measured immediately after the intervention period statistically significant positive effects were found in 22 studies (see Tables 2 and 3), and a statistically significant effect favouring the active control group was identified in a study of auditory memory (Duff et al., 2022). This control was an unvalidated digital cognitive training intervention which was compared to a validated version BrainHQ©. In studies with later follow up measures (Diaz Baquero et al., 2022; Cavallo & Angilletta, 2019; Cheng et al., 2022; Duff et al., 2022; Li et al., 2019; Manenti et al., 2020), positive effects remained but were not statistically significant in two studies (Li et al., 2019; Manenti et al., 2020), and negative effects were no longer evident in one study (Duff et al., 2022). Sustained significant effects were found in two RCTs of high and moderate quality (Cavallo & Angilletta, 2019; Cheng et al., 2022). Participants with Parkinson’s disease and MCI received remote transcranial magnetic stimulation (rTMS) alongside virtual reality cognitive training. They showed sustained improvement in delayed memory three months post intervention compared to those receiving rTMS alone, and sustained improvement in delayed memory and total RBANS scores compared to those receiving neither intervention (Cheng et al., 2022). Participants with early-stage Alzheimer’s who received computerised cognitive training using Branier© showed significant improvement in scores on the digit span test, two syllable word test, story recall and delayed recall, and the Brixton test 12 months post intervention compared to individuals receiving unstructured computer use; however, improvements declined compared to immediately after completing the intervention (Cavallo & Angilletta, 2019). The evidence contributing to this synthesis was mostly moderate quality (23%–87%) and mainly RCTs (see supplemental material). Ten studies, including nine of the RCTs, were low quality, lacking details of blinding and concealment, justification of sample size, and clarity regarding reliability of outcome measures (Amjad et al., 2019; Chen et al., 2022; Han et al., 2020; Jirayucharoensak et al., 2019; Li et al., 2019; Marin et al., 2022; Park, Yun, et al., 2020; Phatak et al., 2021; Sautter et al., 2021; Yang et al., 2022). Therefore, the certainty of whether digital interventions can improve cognition is low.

##### Health and wellbeing

Twenty-nine articles explored health and wellbeing, data were included from 18 using it as the primary outcome or not specifying that it was not the primary outcome (see Tables 2 and 3). Thirty-four outcome effects were identified, and positive effects were found across measures of emotional wellbeing, physical activity, and health. Large effect sizes favouring digital interventions were reported between groups in two studies using the Geriatric Depression Scale (Sautter et al., 2021) and the Pleasant Activities List (Kerkhof et al., 2022); and a small effect size was found using within group analysis on measures of walking abilities (Thapa et al., 2020). However, these didn’t reach statistical significance. Significant improvements immediately post intervention were found using within group analyses in four studies (Bojan et al., 2021; Petersen et al., 2020; Singh et al., 2022; Zhu et al., 2022) and between groups in four studies (Micarelli et al., 2019; Sautter et al., 2021; Shyu et al., 2022; Thapa et al., 2020) using measures of depression, positive and negative affect, stress, mindfulness awareness, walking ability, dexterity, grip strength, dizziness handicap, and balance confidence. 27% of RCT evidence contributing to this synthesis was of low quality (Sautter et al., 2021; Singh et al., 2022; Yang et al., 2022) and causal certainty is low. Quality was low due to unclear blinding, no group concealment, differences between groups at baseline and because groups were not treated identically. Additionally, a significant effect favouring the physical training control group was found on depressive symptoms in one non-randomised control trial of moderate quality (Tsolaki et al., 2020). This intervention was not digital, and therefore questions are raised as to whether a digital method of delivery is more effective than in person. Given the low quality of this evidence and conflicting findings, the certainty regarding whether digital interventions improve health and wellbeing is low.

##### Social relationships

Seven studies explored social relationships, data were included from five measuring social relationships as the primary outcome or not specifying that it was not the primary outcome (see Tables 2 and 3). Six outcome effects were identified showing positive effects in mutuality (positive quality of relationship between carer and care recipient), and social participation. Most did not reach statistical significance, except two using the Personal and Social Performance Scale, Quality of Carer Patient Relationship scale, and Mutuality scale (Chen et al., 2022; Laird et al., 2018). Both used within group analysis measured pre and post intervention, neither had a control group or further follow up, thus the certainty regarding whether digital interventions caused this effect and whether this was sustained is low. Although the quality of studies contributing to this synthesis was mostly high (Kerkhof et al., 2022; Laird et al., 2018; Rai, Schneider, & Orrell, 2021) two were low quality (Beentjes, Neal, et al., 2020; Chen et al., 2022). Most were non-randomised control designs (80%), and only one compared the intervention to a no treatment control group (Rai, Schneider, & Orrell, 2021). Quality was commonly reduced due to lack of detail on randomisation, concealment, blinding processes, and difficulty determining whether both groups were treated identically. Thus, it is difficult to determine whether improvements were the result of digital interventions, or confounding variables and therefore the certainty of this finding is low.

### Qualitative synthesis

#### Study characteristics

Study designs consisted of mixed methods (Beentjes, Kerkhof, et al., 2020; Beishon et al., 2021; Berenbaum et al., 2020; Bogza et al., 2020; Kerkhof et al., 2022; König et al., 2022; Mattos et al., 2021; Rai et al., 2021a, 2021b; Sheehy et al., 2022) and qualitative papers (Berrett et al., 2022; Bielsten et al., 2020; Critten & Kucirkova, 2019; Flynn et al., 2022; Hicks et al., 2019; Ryan et al., 2020; Smith et al., 2020; Øksnebjerg et al., 2019). Five articles described their methods as aligning with participatory action research (Beentjes, Neal, et al., 2020; Berenbaum et al., 2020; Critten & Kucirkova, 2019; Flynn et al., 2022; Hicks and Innes, 2019). Four articles did not report their location (Beishon et al., 2021; Flynn et al., 2022; Mattos et al., 2021; Smith et al., 2020), the rest were in Europe (Beentjes, Kerkhof, et al., 2020; Bielsten et al., 2020; Critten & Kucirkova, 2019; Hicks et al., 2019; Kerkhof et al., 2022; König et al., 2022; Rai, Griffiths, et al., 2021; Ryan et al., 2020; Øksnebjerg et al., 2019), Israel (Berenbaum et al., 2020), Australia (Berrett et al., 2022), and Canada (Sheehy et al., 2022). The duration of interventions ranged between <1 day to 18 months, with most lasting 12 weeks.

Evidence from 295 participants was included in the qualitative synthesis comprising of data from individuals with unspecified, or combined dementia diagnoses (*n* = 184), mild cognitive impairment (*n* = 44), and individuals with dementia or mild cognitive impairment (*n* = 67). In some cases, family members supporting the person living with dementia to use the digital intervention were included in the synthesis when interviewed with the person living with dementia. The participants ages were reported in 13 studies, ranging between 41–97 years, the mean age was 74 years (Beentjes, Kerkhof, et al., 2020; Beilsten et al., 2021; Berenbaum et al., 2020; Bogza et al., 2020; Critten & Kucirkova, 2019; Flynn et al., 2022; Hicks et al., 2019; König et al., 2022; Mattos et al., 2021; Ryan et al., 2020; Sheehy et al., 2022; Smith et al., 2020; Øksnebjerg et al., 2019). Seven studies documented ethnicity, representing eastern and western ethnicities (Beishon et al., 2021; Berenbaum et al., 2020; Bogza et al., 2020; Hicks et al., 2019; Rai et al., 2021a, 2021b; Øksnebjerg et al., 2019).

Digital interventions were categorised by their software and hardware. Several studies used various ‘off the shelf’ devices (Berrett et al., 2022; Hicks et al., 2019; Øksnebjerg et al., 2019; Smith et al., 2020). Two digital interventions were described within multiple studies: FindMyApps (Beentjes, Kerkhof, et al., 2020; Kerkhof et al., 2022), an application to assist people to download dementia friendly tablet apps, and iCST (Rai et al., 2021a, 2021b), an individual cognitive stimulation application. Other digital interventions were categorised as computerised cognitive training (Beishon et al., 2021; Berenbaum et al., 2020), virtual reality games (Sheehy et al., 2022) and virtual reality experiences (Flynn et al., 2022), assistive technologies (König et al., 2022), reminiscence apps (Critten & Kucirkova, 2019), self-management apps (Bielsten et al., 2020), a web-based decision-making tool (Bogza et al., 2020), and online cognitive behavioural therapy for insomnia (Mattos et al., 2021).

In general, data were from interviews, focus groups, and observations, and analysed using thematic analysis (61%) to produce themes summarising participants experiences supplemented by participant quotes. All articles gathered data from carers and people living with dementia except one observational study (Smith et al., 2020) which only gathered data from people living with dementia. Most (61%) used dyadic interviews or focus groups, two conducted separate interviews but combined data during analyses (Flynn et al., 2022; Ryan et al., 2020). It was unclear how data were collected in three articles; however, carer and participant data were analysed together (Beentjes, Kerkhof, et al., 2020; Berenbaum et al., 2020; Konig et al., 2022).

#### Experiences of using digital interventions

A total of 139 findings related to experience of using digital interventions were extracted and synthesised to create thirty review findings, eleven categories and four synthesised findings. These are presented in Table 5 and described below.

**Table 5.** Meta-aggregation of qualitative findings.

Synthesised finding 1	Categories	Findings	Supporting illustrations
**Tailored support is needed to ensure digital interventions are accessible to people with dementia.**	**Category 1:** Supporting people with dementia to learn and use digital interventions is essential for creating accessibility.	People with dementia and carers acknowledge they need support to learn and use digital interventions	“Searching independently [for apps] was not possible’’ (Beentjes, Kerkhof, et al., 2020, *caregiver*)
		Supporting the learning process and use of digital interventions is needed to empower people with dementia to use digital interventions	‘‘She [caregiver] has to help me. I couldn’t do it myself.’’ (Beentjes, Neal, et al., 2020, *person with dementia*)
			“I couldn’t do it myself, someone had to be around” (Kerkhof et al., 2022, person with dementia)
			“Downloading apps was difficult, we did this mostly together, he never did it alone.” (Kerkhof et al., 2022, caregiver)
			“I was pleased with my abilities, it proved that I was, there was quite a lot there that I could cope with” (Beishon et al., 2021, person with dementia)
			“The training was a turning point for me, that was the turning point for me” (Ryan et al., 2020, *person with dementia*)
			“Just interesting to see how people who initially didn’t look like they were that involved in it…even capable of being involved with it … took to it…So just to see that obviously it makes me look at things differently…and I don’t now automatically assume that they are (unable to participate), you know, probably give them more time than maybe I would have before.” (Hicks et al., 2019, *Volunteer*)
		Unsuccessful attempts to support learning and use lead to disengagement	“It takes a lot of effort to learn and [the participant] didn’t have patience for that.” (Beentjes, Kerkhof, et al., 2020, *person with dementia*)
			Not all participants that disengaged did so every time the technology was presented which suggests in some instances that disengagement was the product of unsuccessful scaffolding and support at that particular time. It could also be a consideration that the condition was being experienced differently between sessions, on different days and the individual felt little desire to engage with the technology when compared with previous sessions (Smith et al., 2020, *observation)*
	**Category 2:** People living with dementia have different needs and preferences for how they would like to be supported to use digital interventions	Some people with dementia and carers prefer and require structured support in the form of instructions.	“One male living with dementia reported finding the instructions useful ‘anything that helps make life easier’” (Flynn et al., 2022*, person with dementia*)
			Others asked the research assistants for help with specific tasks, or with their general confusion. Some reread the instructions out loud or to themselves and consciously contemplated the instructions. For example, sometimes they counted out loud to keep track of what they were doing. (Berenbaum et al., 2020, *Observations*)
			“I just need [..] normal instructions” (Flynn et al., 2022, *caregiver*)
		Some people with dementia prefer time and space to explore learning independently.	“The wee iPad definitely. The iPad and getting on to it. There’s a lot to learn on it yet. I’ve plenty of time to do it. It’s keeping the head thinking.” (Ryan et al., 2020, *person with dementia*)
			Observations indicated the use of various learning techniques in order to complete the game. Some of the PwD used the trial and error method. It was sometimes evident that they used a self-correcting mechanism when they felt that their first attempt was incorrect. (Berenbaum et al., 2020, *Observations*)
			“I just keep on [working] with it until I succeed” (Kerkhof et al., 2022, person with dementia)
		Some people with dementia require and prefer more hands-on support in the form of physical assistance	“It is really easy to take the photos as long as someone holds this [the iPad]. I Liked choosing the photos and thinking up what to write underneath.” (Critten & Kucirkova, 2019, *person with dementia*)
			Robert had good coordination, but was concerned he might drop the iPad. He was able to take photos when somebody held the iPad for him. (Critten & Kucirkova, 2019, *Observation*)
	**Category 3:** The relationship between the person living with dementia and the individual providing support is important	Some people with dementia prefer to be supported by close familial individuals	“Do you think that I could do this activity with anybody and I think the answer to that would be no.” (Rai, Prasetya, et al., 2021, *person with dementia*)
			Three people living with dementia verbalized that the presence of the caregiver was useful while they were using VR in real-time (Flynn et al., 2022, *Field Note*)
		Some people with dementia prefer to be supported by individuals they perceive as knowledgeable	“No, I go upstairs and ask help from my grandchildren. They are expert on this. I Try to ask for help first rather than switch off.” (Rai, Griffiths, et al., 2021, *person with dementia*)
			“It’s just because you [researcher] have a good knowledge about it and what sort of things people do right or wrong” (Flynn et al., 2022*, person with dementia*)
**Synthesised finding 2**	Categories	Findings	Supporting illustrations
**Digital interventions must have a purpose to be desirable to people living with dementia and their caregivers.**	**Category 1:** People with dementia and their caregiver’s pre-existing attitudes about technology influence their desire and perceived ability to engage in digital interventions.	Familiarity with technology is perceived as an important facilitator for being able to use digital interventions.	“[He] already had a smartphone so [he] was already familiar with technology.” (Beentjes, Kerkhof, et al., 2020, caregiver)
			“I think he managed very well, of course we practise on a regular basis, but he was already very experienced using a computer and a tablet” (Kerkhof et al., 2022, *caregiver*)
			“For me it would be a tool that I’m using every day of the week. If you was given a new laptop, how would you feel, because it’s now a different system you would feel lost I’d tell ya!” (Rai, Griffiths, et al., 2021, *Person with dementia*)
		Carer’s and people with dementia’s opinions and feelings towards technology influence whether they desire and accept digital interventions.	“Before (being) diagnosed with dementia, my mother always refused to use gadgets. We try to convince her to use mobile phone, but she did not want it. So, since then she rarely used technology and even now, when she has dementia, she never wants to use it**”** (Rai, Prasetya, et al., 2021, *caregiver*)
			“I would put the whole thing down to me. I’m not accepting of it. I wasn’t maybe doing as much as I should have been doing.” (Ryan et al., 2020, *caregiver*)
			“I love technology. It is wonderful to use this small computer and use the internet.” (Critten & Kucirkova, 2019, *person with dementia*)
		How people with dementia and carers appraise their ability to use technology influences their decisions to try digital interventions.	“I would not be very techie. However, for the knowledge that I do have about downloading an app, I think I would be able to manage that [. . . ] and then there’ll be instructions with it so, I think I would be okay to manage it.” (Flynn et al., 2022, *caregiver*)
			“I think it’s a big thing for older people. You have got to get them realising that they are actually capable of doing this.” (Rai, Griffiths, et al., 2021, *person with dementia*)
	**Category 2:** Digital interventions need to be useful to motivate people with dementia and their carers to use them	Motivation to use digital interventions is driven by a need to stimulate, support and keep the brain active	“I want to know what we as a caregiver can do for our person with dementia, such as a game, to stimulate their brain so they can interact, play and communicate more.” (Rai, Prasetya, et al., 2021, *caregiver*)
			“The reason we participated in this study was to try and stimulate my husband’s brain. This is because he is showing less initiative and prefers to watch television all the time. So, it would be good to activate his brain more to slow down the dementia process. And it worked.” (Kerkhof et al., 2022, *caregiver*)
			“I think anything that you do, you do together that stimulates the brain at any time, even if it’s a short space of time, I’ll have a go at it.” (Rai, Griffiths, et al., 2021, *caregiver*)
		Digital interventions that fulfil a purpose are perceived as useful to people with dementia and caregivers	‘‘Difficult, we can do little with FindMyApps. [We] just use [our] telephone or laptop’’ (Beentjes, Kerkhof, et al., 2020, caregiver)
			“they’ve got to be explaining what you’re supposed to be doing and a purpose other than the obvious thing of identifying sea urchins, sea animals, that’s obvious what that’s about but what is that actually helping? To get some feedback on my brain, I am coping, how I’m doing?” (Beishon et al., 2021, *person with dementia*)
			“I found some of the exercises pointless” (Sheehy et al., 2022, person with dementia)
**Synthesised finding 3**	Categories	Findings	Supporting illustrations
**Digital intervention designs need to be tailored to the needs of people with dementia for successful incorporation into their lifestyle.**	**Category 1:** Symptoms associated with dementia and aging impact on the feasibility of people with dementia successfully using digital interventions.	Symptoms and difficulties related to dementia progression impact on the feasibility of people with dementia being able to successfully use digital interventions	“But I kept having to remind him [the patient], you know let’s get the puzzles tonight or, and then once we got going then it was fine, he’d just forget” (Beishon et al., 2021, *caregiver*)
			“I do have a memory problem that’s already been identified by a neurologist, that was difficult for me,” and “. . . the problems I’m having most likely ha[ve] to do with my memory issues.” (Mattos et al., 2021, *person with dementia*)
			“Motivation is something that’s really gone, you know, motivating [patient] to do a lot of things isn’t as easy as it used to be” (Beishon et al., 2021, *caregiver*)
		Symptoms and difficulties related to aging influence the way in which people with dementia can use digital interventions	“Because my eyesight is no longer a hundred percent, I’m quite happy on a computer. I’m more at home doing it on the computer, the small keys on my mobile, you know” (Beishon et al., 2021, *person with dementia*)
			“Oh, sometimes when I want to type something, a different letter pops out. So I have to erase all and start again.” (Rai, Prasetya, et al., 2021, *person with dementia*)
			People living with dementia who easily managed the controllers expressed a preference for greater interactivity and free exploration to satisfy their curious nature and increase their agency [PwD 1, 6, 9]. Such participants had no physical difficulties and were observed to have good standing or sitting balance. (Flynn et al., 2022, *Observation*)
	**Category 2:** Convenient, good quality and easy to use digital intervention designs are important to increase the usability of digital interventions.	The design of the software is an important facilitator in whether digital intervention are seen as usable	“It’s very user friendly. It is not difficult at all. I Am not at all technical with computers and tablets, but even I understand this.” (Kerkhof et al., 2022, *caregiver*)
			“it’s very scientific. I don’t identify with the text.” (Bogza et al., 2020, *person with dementia*)
			“It was simple. It was nice and easy… the wee app and the photographs, it was easy to do. You know, I think that, because the programme was so simple, do you know what I mean, if it had’ve been something more complicated I think we would’ve lost interest” (Ryan et al., 2020, *caregiver*)
			“The repetition of the puzzles as we became more familiar made it smoother and less restrained but at the start it was a bit frustrating” (Beishon et al., 2021; caregiver)
		The design of the hardware is important to facilitate convivence, ease and usability	“It makes it seem like it’s a more academic grown-up activity. Some of these games you would think are suitable for children, but if you’re doing it on a computer, it looks more grown up somehow.” (Rai, Schneider, & Orrell, 2021, *caregiver*)
			Rose: That monitor is fantastic, so I just entre whatever is happening
			Bob: It’s not in the way
			Rose: It’s really good, and you can read it from a distance. It’s really good, we’re really happy with that (Berrett et al., 2022, *caregiver and person with dementia*)
			“I think it’s wonderful because it’s so small and so compact. It’s something that you can give a little wipe and clean and it looks good and everything is stored in there, all that information that you want about your family history from when you were growing up, right through to now …“ (Ryan et al., 2020, *person with dementia*)
			“Well, the thing about it, you have it on your knee, and you can sit and just go through it, you know … Whereas, if it’s an album, usually they’re stuck in a drawer or something, and you wouldn’t be bothered going to look for them.” (Ryan et al., 2020, *person with dementia*)
		Technical difficulties and faults negatively impact on the usability of digital interventions	“Frustrating overall because often the computer got hung up or I had to repeat some things when I was rushing through” (Mattos et al., 2021, *person with dementia*)
			“We had to go through a few steps to get the thing started each morning. (The iPT) comment that that shouldn’t have been necessary but in fact it as. So that is some kind of technical glitch that we ran into…” (Sheehy et al., 2022, *caregiver*)
			“The frog leap one I found that really kind of frustrating because even though I felt that I was doing what I was supposed to, it wasn’t picking up and I had to keep repeating it over and over again, and then I felt that was soul destroying for me because I kind of felt that I was following but effectively it was saying that I wasn’t” (Beishon et al., 2021, *Person with MCI*)
			All participants expressed frustration with the need to set up and maintain software, for example, updating apps. (Øksnebjerg et al., 2019, *Field note*s)
		Good quality interventions enhance the usability and positive experience of using digital interventions	“Particularly the quality of the sound you get versus the smaller ipad, which is significant for me, because it actually makes it pleasurable to listen to, otherwise it’s awful because you can’t get the range, so that’s nice. I don’t know whether the neighbours like it all the time because it’s quite often outside (laughter). And I can hear it from the street out front” (Berrett et al., 2022, *person with dementia*)
			“Marvellous… [VR] so realistic” (Flynn et al., 2022, *person with dementia*)
	**Category 3:** Integrating digital interventions into lives of people with dementia is challenging and require routine and structure to be successful.	Using digital interventions is not always seen as a priority to people with dementia or carers	“I can’t get it off the ground, my husband was always against using laptops and computers. At the moment I have to deal with all kinds of family issues and in combination with my energy level necessary to take care of my husband this makes it impossible. It is just a matter of lack of time.” (Kerkhof et al., 2022, *caregiver*)
			“Yes, but it’s having the time though…That’s the problem with it.!” (Rai et al., 2021, *person with dementia*)
		Incorporating the use of digital interventions into routine is seen as a facilitator to maintain its use	“When I first came here and I went home I was doing it religiously, but then it slipped my mind and then I found it difficult to get it back in the routine” (Beishon et al., 2021, *person with dementia*)
			“It’s a matter of establishing a routine. If that routine is gone, you have to relearn it bit by bit” (Kerkhof et al., 2022, *person with dementia*)
			“However, at the end the stimulation is very important because it can be a habit if we do it repeatedly” (Rai et al., 2021, *caregiver*)
			Some participants believed that SHUTi OASIS provided structure to their daily routine (*n* = 3), requiring discipline to enter daily sleep dairies, leading to feeling “empowered.” (Mattos et al., 2021, *Author’s Statement*)
**Synthesised finding 4**	Categories	Findings	Supporting illustrations
**People living with dementia can benefit from digital interventions.**	**Category 1:** Digital interventions provide people with dementia opportunities to connect with others and their interests which facilitates enjoyment	Digital interventions provide opportunities for people with dementia to connect to their peers and community	“I think a lot of it is very good … and you find, good lord, what a funny game this is. And, it’s great fun, and you can see it, and all of them respond [the men], I respond, I don’t always win but it’s great!” (Hicks et al., 2019, *person with dementia*)
			“I have a group of ex co-workers from the office (on WhatsApp) and there are many members, so we share information or news such as health articles.” (Rai, Prasetya, et al., 2021, *person with dementia*)
			“Yes, I think [that my life has changed]. It hasn’t changed a lot, but I do think that it changes you. You know more, you hear more, and you see more, and your social environment is different. When I look at my sister, well, her world is very small. And a tablet can make it bigger” (Kerkhof et al., 2022, *person with dementia*)
		Digital interventions provide opportunities for connecting with interests and facilitate enjoyment	“I could see him lighten up, do you know what I mean, when he’d see pictures or music or something that he knew, do you know what I mean. I Could see him interested in it which I felt was, he doesn’t have much interest in anything really” (Ryan et al., 2020, *caregiver*)
			“I loved the golf game … I can’t go out and play on a course anymore. I don’t have the money or equipment and … I’m not physically able to now but I love it … and I played it and I hit a good shot occasionally. About once every three weeks!” (Hicks et al., 2019, *person with dementia*)
			“Yes, and I love to read regarding health. I Spend my time reading rather than just sleeping. I Do not feel comfortable if I do nothing” (Rai, Prasetya, et al., 2021, *person with dementia*)
		Digital interventions can be a tool to scaffold social interaction and conversation between users	“We talked about conflicts and stress, the fourth chapter. And how we experience that […] I have not dared before […], I have occasionally said that I am sorry because I am irritated and yes, it is […] yes, I feel calmer in some way […] now (C3)” (Bielsten et al., 2020, *caregiver*)
			The iPad to locate areas of East London where he grew up. We had an interesting chat about this and he was able to find his old street using Street View…He discussed the places where he had worked and showed me the streets he had frequented. He repeatedly commented that it had all changed with all the houses that had sprung up everywhere. (Hicks et al., 2019, *Observation*)
		Digital interventions can foster feelings of closeness and connection within relationships	“I think he’s more understanding… Well, I think it’s talking about it and accepting the way that I am, you know, but trying not to think about it. I don’t let it be the main thing in my mind that my memories not good.” (Ryan et al., 2020, *person with dementia*)
			“We spent whole evenings with music playing, singing along with old songs from the past via the ‘dementie en herinneringen app’ (dementia and memories app)].” (Beentjes, Kerkhof et al., 2020, *person with dementia*)
			“To spend more time together really I suppose. Other than that we do different things when we’re not together so at least you spend more time together yes.” (Rai, Griffiths, et al., 2021, *person with dementia*)
			“The app handles a lot of issues related to couple relationship and it is without doubt that you are two with the disease in this disease” (Bielsten et al., 2020, caregiver)
	**Category 2:** People with dementia’s appraisal of their progress and abilities of using digital interventions can positively or negatively influence their mood and experience	Digital interventions that provide opportunities to monitor progress can enhance enjoyment if progress is appraised positively	“To see that I was actually achieving something, getting better at certain things than I started off I suppose that’s really the ideal thing is to see an achievement isn’t int?” (Beishon et al., 2021, *person with dementia*)
			“Well it made him do a little bit of exercise, but it also boosted his morale in as much as he felt he was achieving, he felt that he was you know winning the game” (Hicks et al., 2019, *caregiver*)
			“As far as I’m concerned, I come here very glum, fed up and sorry for myself and I go away thinking that I can still do something even if it’s something silly. I Can still do something.” (Hicks et al., 2019*, person with dementia*)
			“The brain games exercised me and that was good, and I think that with some of the games I was doing pretty well and I improved a little bit as the time went on so that’s been good” (Beishon et al., 2021, *person with MCI*)
		Digital interventions that provide opportunities to monitor progress risk having a negative impact on mood if progress is appraised negatively.	Carer: You just couldn’t pass them. Couldn’t pass the exercises could you? So it made you feel a failure that you couldn’t cope with the exercises.
			Patient: I don’t know about that. Or maybe I just didn’t want to. If you like I’ve given up. (Beishon et al., 2021*, caregiver and person with dementia*)
			“As they failed to hit their opponent, they became increasingly frustrated with their performance as well as the volunteer supporting them, with one man shouting: ‘What would you know? How many fights have you been in?’ (Peter, Marching on, RFN session three). During feedback, tel suggested he hadn’t liked the game as it was ‘too unrealistic’ (tel, Marching on, RFN, session three) and consequently the game was never played again.” (Hicks et al., 2019, *Observation*)
			“I think like with the word one, a bit depressed because I thought I should be able to do these and that gets me down because I think it’s hard, I think there’s something wrong with me” (Beishon et al., 2021, *person with dementia*)
	**Category 3:** Positive experiences using digital interventions can have noticeable effects on people with dementia that have the potential for change.	Digital interventions stimulate noticeable changes in people with dementia’s cognition and behaviour	“I think, he was a lot more alert, since he started doing this, since about the second week, than he was before that, before that you spent a lot more time sitting, dozing in your chair, and after that you were doing much more with your guitar, and your amplifier, you were working on, whatever, that stuff. You were more alert.” (Sheehy et al., 2022, *person with dementia*)
			“::: it was a difference in my walking, I couldn’t help but notice it.” (Sheehy et al., 2022, *person with dementia*)
			“I definitely felt that it improved my abilities to think because I had something to think about very hard in those programs” (Beishon et al., 2021, *person with dementia*)
		Positive experiences of using digital interventions have the potential to influence people’s attitudes surrounding their ability to use technology	“It’s a part of my life now. It was something that I never thought that I could use. I didn’t think that I could use that …” (Ryan et al., 2020, *caregiver*)
			“You know, I grew up with pen and paper. But you can live without it nowadays” (Kerkhof et al., 2022, *person with dementia*)
			“Well, I have become more digital. Before, I would have used a paper file” (Kerkhof et al., 2022, *person with dementia*)
			First-hand experience of using a technology probe enhanced people living with dementia and caregivers’ understanding of VR *[PwD 3,8; CG 2, 3, 9]* and addressed their preconceptions *[CG 1–9]*. Some of the people living with dementia and their caregivers reported gaining an “idea of what it was like” [PwD 5] (Smith et al., 2020, author statement)
		Positive experiences using digital interventions have the potential for technology to be in other contexts.	“When she gets visitors, aye, she just loves it, or when something happens, like, when my brother was back, we took the iPad out for dinner … usually mobile phones are banned at the table, and all this, but we were allowed the iPad out for photos and all, and she was trying to take some herself.” (Ryan et al., 2020, *caregiver*)
			“It [the TI] sets you up to use it [technology] at home as well doesn’t it? So that you don’t just use it there but when you come home you can look back on what you did and then continue where you left off.” (Hicks et al., 2019, *person with dementia*)
			“It definitely stimulated his interest, he said ‘I want a tablet of my own. Because when I am sitting outside, I enjoy using it.’ so, we bought a tablet” (Kerkhof et al., 2022, *caregiver*)

##### Synthesised finding 1: Tailored support is needed to ensure digital interventions are accessible to people living with dementia

People living with dementia and their caregivers acknowledge that support to learn and use digital interventions is crucial to empower them (Beentjes, Kerkhof, et al., 2020; Beishon et al., 2021; Hicks et al., 2019; Kerkhof et al., 2022; Ryan et al., 2020), and if not supported, this can lead to disengagement (Beentjes, Neal, et al., 2020; Smith et al., 2020). Support must be tailored to the needs and preferences of people living with dementia, who differ in how they would like to be supported, and who they would like to be supported by. Some prefer to be supported by family (Flynn et al., 2022; Rai et al., 2012b), and others by individuals they feel are knowledgeable about technology (Flynn et al., 2022; Rai et al., 2012a). The level of support ranged from independent exploration (Berehaum et al., 2020; Kerkhof et al., 2022; Ryan et al., 2020), providing instructions (Berenhaum et al., 2020; Flynn et al., 2022), to verbal or physical assistance such as holding devices, or physically guiding participants around devices (Critten & Kucirkova, 2019).

##### Synthesised finding 2: Digital interventions must have a purpose to be desirable to people living with dementia and their caregivers

People living with dementia and their caregivers have pre-existing attitudes towards technology which influence the desirability of using digital interventions. Lack of familiarity with technology (Beentjes, Kerkhof, et al., 2020; Kerkhof et al., 2022; Rai, Griffiths, et al., 2021), negative opinions (Critten & Kucirkova, 2019; Rai, Prasetya, et al., 2021; Ryan et al., 2020) and low skill appraisal (Flynn et al., 2022; Rai, Griffiths, et al., 2021) make digital interventions less desirable. To improve desirability, digital interventions need a purpose to be seen as useful (Beentjes, Kerkhof, et al., 2020; Beishon et al., 2021; Sheehy et al., 2022). Caregivers' desires to stimulate, support, and keep the brain active are powerful purposes for initiating use of digital interventions (Kerkhof et al., 2022; Rai et al., 2021a, 2021b). However, people with dementia also need to find the intervention useful for it to be maintained (Beentjes, Neal, et al., 2020; Beishon et al., 2021; Sheehy et al., 2022).

##### Synthesised finding 3: Digital intervention designs must be tailored to the needs of people living with dementia for successful incorporation into their lifestyle

Symptoms such as apathy and memory difficulties, associated with dementia progression make individuals less likely to, or less able to use digital interventions (Beishon et al., 2021; Mattos et al., 2021). Deterioration of eyesight and physical abilities, associated with normal aging also make digital interventions more difficult to use (Beishon et al., 2021; Flynn et al., 2022; Rai, Prasetya, et al., 2021). There appeared two ways of mitigating these difficulties.

Firstly, designs prioritising simplicity (Beishon et al., 2021; Bogza et al., 2020; Kerkhof et al., 2022; Ryan et al., 2020) and convenience were deemed more usable, and less stigmatising (Berrett et al., 2022; Rai, Griffiths, et al., 2021; Ryan et al., 2020). Additionally, good quality enhanced intervention appeal (Berrett et al., 2022; Flynn et al., 2022). Those that require multiple updates are deemed less usable because they produce technical difficulties making participants frustrated and disengaged (Beishon et al., 2021; Mattos et al., 2021; Sheehy et al., 2022; Øksnebjerg et al., 2019). Difficulties are mitigated when intervention use is successfully incorporated into the routines of people living with dementia (Beishon et al., 2021; Kerkhof et al., 2022; Mattos et al., 2021; Rai, Griffiths, et al., 2021). This is acknowledged as challenging and not always a priority (Kerkhof et al., 2022; Rai, Prasetya, et al., 2021).

##### Synthesised finding 4: People living with dementia can benefit from digital interventions

Digital interventions provide opportunities for connectedness. Their use can scaffold social interaction, stimulate conversations (Bielsten et al., 2020; Hicks et al., 2019), and connect people living with dementia to their peers and community (Hicks et al., 2019; Kerkhof et al., 2022; Rai, Prasetya, et al., 2021). When used with family, digital interventions can foster feelings of closeness (Beentjes, Kerkhof, et al., 2020; Bielsten et al., 2020; Rai, Griffiths, et al., 2021; Ryan et al., 2020). Digital interventions focusing on leisure provide opportunities to connect with hobbies and interests, creating feelings of enjoyment (Hicks et al., 2019; Rai, Prasetya, et al., 2021; Ryan et al., 2020). However, enjoyment depends on how ability and progress is appraised during use. When people appraise their progress and ability as positive, this produces feelings of achievement (Beishon et al., 2021; Hicks et al., 2019), whereas poor progress leads to frustration and disengagement (Beishon et al., 2021; Hicks et al., 2019).

For some, noticeable changes in cognition and behaviour reinforce positive experiences of use (Beishon et al., 2021; Sheehy et al., 2022). Appraising digital interventions as a positive experience has the potential to improve people’s perception of their technology skills (Kerkhof et al., 2022; Ryan et al., 2020; Smith et al., 2020), and empower people to use technology in different settings and for different purposes (Hicks et al., 2019; Ryan et al., 2020).

## Integration of quantitative and qualitative syntheses

**Figure 3.**
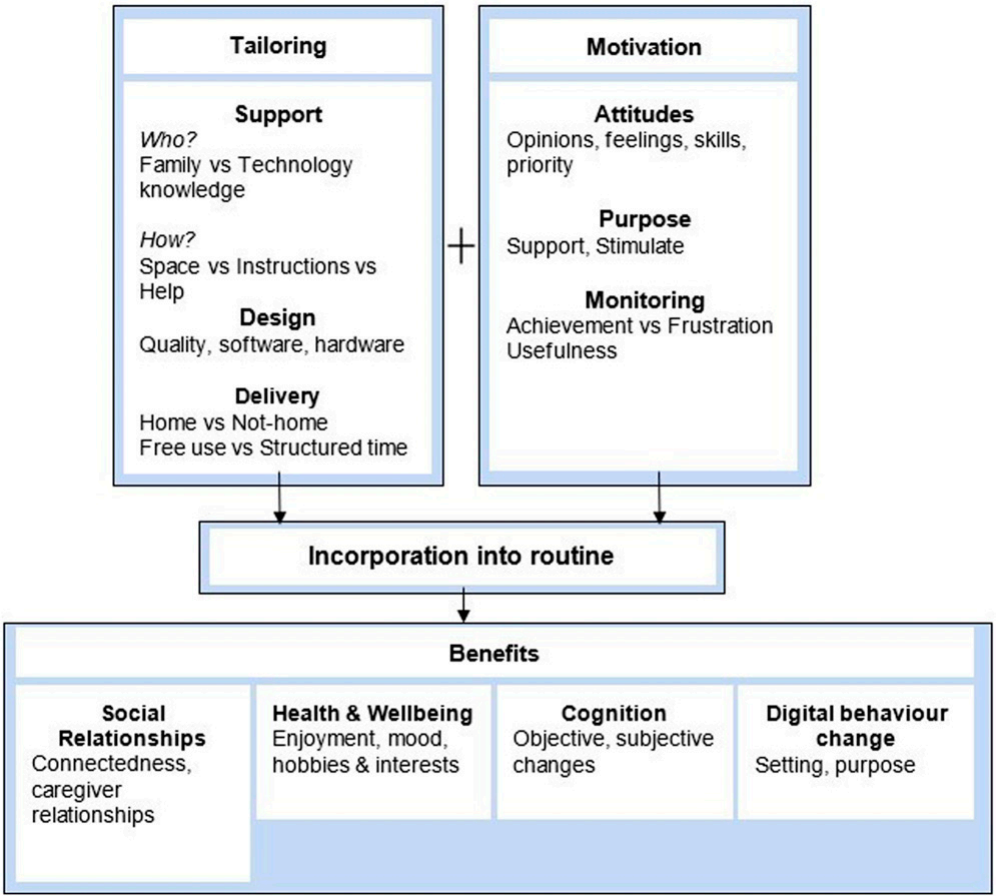
Integration of evidence summarising benefits of using digital interventions and how these benefits might be achieved.

The integration of evidence reveals how people living with dementia can benefit from using digital interventions and how delivery might be optimised (Figure 3).

### Benefits to digital intervention use

Benefits were identified qualitatively and quantitatively in social relationships, health and wellbeing, and cognition. Additionally, changing where and with whom people living with dementia used digital technologies was identified in the qualitative synthesis.

Socially, digital interventions contribute to social interaction and integration by scaffolding conversation and connecting people living with dementia to their peers and community (Bielsten et al., 2020; Hicks et al., 2019; Rai, Griffiths, et al., 2021; Smith et al., 2020). Collaborative use was described as fostering feelings of closeness (Beentjes, Neal, et al., 2020; Bielsten et al., 2020; Rai, Griffiths, et al., 2021; Ryan et al., 2020), shown in the quantitative data as improvements in measures of caregiver relationships and mutuality (Chen et al., 2022; Laird et al., 2018).

Interestingly, leisure was rarely a focus for interventions, yet enjoyment was commonly described qualitatively as a benefit (Flynn et al., 2022; Hicks et al., 2019; Rai, Prasetya, et al., 2021; Ryan et al., 2020; Sheehy et al., 2022). Quantitatively, improvements were seen on measures of mood and positive affect indicating enjoyment (Kerkhof et al., 2022; Sautter et al., 2021; Shyu et al., 2022).

Changes in physical activity, alertness, and cognition are described qualitatively (Beishon et al., 2021; Flynn et al., 2022; Sheehy et al., 2022). These are consistent with improvements on quantitative measures of physical activity and cognitive tests following the intervention (Micarelli et al., 2019; Petersen et al., 2020; Thapa et al., 2020).

Changes to how people used digital technologies involves people feeling more motivated to use the technologies outside of the research context. These individuals used the interventions and other technologies for new purposes in different locations after taking part in studies Hicks et al. (2019); Kerkhof et al. (2022); Ryan et al. (2020). This was not featured in quantitative papers, as it’s not a variable measured numerically but indicates that positive experiences might contribute to making future interventions and technologies more desirable.

### Optimising digital intervention delivery

A lack of reporting on how digital interventions were provided to participants means it is difficult to conclude the most effective way of providing digital interventions. For example, 39% of quantitative articles did not report either the location of intervention use, the method of intervention delivery, or the level of support given to use the intervention. From available information, structured digital interventions (those requiring a specified amount of use per day/week) provided at home more frequently produce positive effects. These interventions made up 29% of studies reporting location and requirements of intervention use, of which 73% demonstrated positive outcomes. Additionally, evidence from one qualitative study describes home-based digital interventions as more desirable because participants did not have to go out (Ryan et al., 2020). Further qualitative evidence suggests incorporation into routine is key to counteract barriers related to memory difficulties (Beishon et al., 2021; Kerkhof et al., 2022; Mattos et al., 2021; Rai, Schneider, & Orrell, 2021). Therefore, interventions with structured requirements for use, provided in the home environment may be more easily incorporated into routines because they are more convenient. This is important as people living with dementia require support to learn and use interventions (Beentjes, Kerkhof, et al., 2020; Critten & Kucirkova, 2019; Flynn et al., 2022; Kerkhof et al., 2022; Rai, Griffiths, et al., 2021), often provided by carer’s (Beishon et al., 2021; Flynn et al., 2022; Kerkhof et al., 2022), and therefore, the convenience of having interventions in the home may mediate instances where they are not seen as a priority because they require less time and effort.

## Discussion

People living with dementia or mild cognitive impairment benefit from digital interventions, particularly those targeting cognition, health and wellbeing, and social relationships. Other benefits include enjoyment from intervention use and continuing to use technologies outside of the research parameters.

To gain benefits, individuals need to be motivated, otherwise interventions are not seen as a priority and forgotten. Motivation often comes from carers who describe a need to stimulate and support, but motivation is maintained when people living with dementia feel the intervention is useful.

People living with dementia require support to use digital interventions, which often comes from carers or people knowledgeable about technology. Also, interventions used at home which have specified requirements to how long they are used per day/week may be more likely to produce beneficial outcomes. The frequency and intensity of intervention use does not improve quantitative outcomes, and so this delivery method is likely effective because it provides a convenient and flexible way to incorporate interventions into routines.

The benefits of using digital interventions found in this review are consistent with previous reviews exploring effectiveness of specific interventions. For example, touchscreen technologies and digital cognitive training improved the psychological wellbeing, cognition, social interaction, and relationships of people living with dementia (Hill et al., 2017; Pang et al., 2021; Tyack & Camic, 2017). Qualitative reviews corroborate this, evidencing benefits in the physical, mental, and social health of people with dementia using mobile health applications (Brown & O'Connor, 2020). Despite this, we cannot conclude with certainty that digital interventions are effective in improving cognition, health and wellbeing, and social interaction. This is because in depth statistical comparisons were impossible due to there being huge variation in how quality of life was conceptualised by authors, and how outcomes were measured. Additionally, the general quality of available quantitative evidence was inconsistent and low further reducing the certainty of benefits. This is important given that most included studies also only explored short term benefits, and thus it also cannot be concluded that the benefits that were identified are sustained. Similar uncertainty is acknowledged elsewhere, where low quality evidence, small sample sizes, and inconsistencies between technologies and designs mean that other reviews have also failed to provide convincing evidence of digital intervention effectiveness (Gates et al., 2019; Knapp et al., 2022; Topo, 2009; Van der Roest et al., 2017).

The qualitative review findings are largely consistent with theoretical models of adaption, specifically the environment-person fit (E-P fit) model’s notion that a good fit between a person’s competencies and environment is required for digital interventions to be desirable, accessible, and useful (Lawton & Nahemow, 1973). For example, our findings show that declines in cognition and poor skill appraisal reduce technology competence, leading to poor E-P fit, and non-adoption of digital interventions. According to our findings one method to improve this fit is by providing support. There is much evidence consistent with our finding that people living with dementia require support to use digital interventions (Evans et al., 2022; Gibson et al., 2015; Lariviere et al., 2021; Smith and Mountain, 2012). Previous frameworks have, like this review, identified that support comes from carers, or people knowledgeable regarding technology (Evans et al., 2022); without support this review found participants were likely to disengage.

The qualitative review findings are also consistent with the concepts described in the unified theory of acceptance technology (UTAUT) (Marikyan & Papagiannidis, 2023). This theory may explain our two novel findings. Firstly, we found that positive experiences using digital interventions resulted in participants using the technologies outside of the study parameters, such as with different people or in different settings (Hicks et al., 2019; Kerkhof et al., 2022; Ryan et al., 2020). Older adult literature describes gaining knowledge as influential for older adult technology use (Nygard & Starkhammar, 2007). For example, digital inclusion classes indicate that individuals learning to use technology show desire to learn about other technologies and this level of desire was mediated by positive experiences, with positive experiences being associated with a desire to learn new technologies (Betts et al., 2019). Aligning with the UTAUT (Marikyan & Papagiannidis, 2023), this is likely because gaining knowledge through classes improves performance expectance (predicted usefulness) and effort expectance (ease of use). In view of this, it is possible that a similar process is established through taking part in digital intervention research. This means that by learning technology skills through research experiences, people with dementia might adapt better to technology environments outside of study parameters.

Secondly, the review found that interventions provided at home, requiring structured frequency of use, were more likely to produce benefits. Like us, Pang and colleagues’ (2021) found no differences in effectiveness outcomes based on intervention length or intensity, suggesting that the delivery method is important for other reasons. Based on the E-P fit model and UTAT (Lawton & Nahemow, 1973; Marikyan & Papagiannidis, 2023) this method of delivery may be effective because it reduces environmental pressures by facilitating a convenient way to incorporate interventions into routines, a common strategy to aid memory (Cavanaugh et al., 1983; Ferland et al., 2013; White, 2023). Like others, we found carers were a key factor in this incorporation (Evans et al., 2022; Gibson et al., 2015; Lariviere et al., 2021; Smith and Mountain, 2012). Therefore, it is possible that barriers such as being too busy or interventions being too time consuming (Brown & O'Connor, 2020; McAllister et al., 2020), might be mitigated by home-based interventions which are more convenient to carer’s (Christie et al., 2018) because these reduce added burden (Arthanat et al., 2020).

### Limitations

Several limitations are applicable to this review, firstly many different study designs and outcomes were included meaning effectiveness was determined by vote counting the direction of effect (McKenzie et al., 2023). This is acceptable in Cochrane guidance (McKenzie et al., 2023) but limited because it can only determine the presence of an effect, not the magnitude.

Secondly, this review includes samples of people living with dementia, mild cognitive impairment, and probable Alzheimer’s disease because many studies used mixed samples, thus, to avoid excluding data, all are included. Therefore, findings may be more applicable to individuals in the prodromal or milder stages of dementia, as a larger proportion of the review sample fit this category. Previous literature shows digital technologies may be more beneficial to individuals with mild cognitive impairment than people living with dementia (Neal et al., 2021), which might explain the positive effects found. The findings on effectiveness should therefore be applied to other stages of dementia with caution.

Thirdly, most studies in this review explored digital intervention effects at the end of the intervention period, thus describing short-term benefits. Long term benefits have rarely been explored however, one review showed benefits of engaging in digital interventions were not evident at 12 months (Gates et al., 2020). Therefore, the longer-term sustainability of digital interventions is unknown.

Finally, poor methodological reporting means 20% of studies were of low quality thus, findings are low in certainty. The addition of qualitative data to the effectiveness synthesis adds some certainty, however, the trustworthiness of qualitative data is not graded. This lack of consistent high-quality evidence is not uncommon among reviews exploring digital technologies (Neal et al., 2021; Van der Roest et al., 2017), however, findings should be interpreted with risks of bias in mind because we cannot conclude with certainty that effectiveness of digital interventions is established.

### Implications and recommendations

Digital interventions in dementia are popular reflected by the large proportion of pilot studies identified. Despite this, this review demonstrates little progress has been made making these real-world applicable, potentially due to researchers’ enthusiasm to invent rather than provide good quality evidence of effectiveness (Knapp et al., 2022). Previous reviews have also not provided convincing evidence because of the many varieties of technology and design being produced (Topo, 2009; Van der Roest et al., 2017). For example, this review describes 28 types of digital cognitive training programmes, despite well established, trademarked versions being available for testing (Kong, 2020). Although 29 of these were RCTs, only 32% were deemed high quality; many were feasibility stage, thus unready for large scale effectiveness testing and not real-world applicable. Further high-quality, large-scale research is needed to determine whether digital interventions are effective.

This review provides a summary of interventions already developed mapped on to the quality-of life domains they target. Consistent with others, the results show developments have mostly focused on improving cognition (Dequanter et al., 2021), however given that the quantitative benefits here remain inconclusive, alternative outcomes should be explored. Personal outcomes such as social relationships and self-concept remain underexplored (Neal et al., 2021). Compared to previous reviews, progress has been made developing digital reminiscence opportunities for community dwelling people living with dementia, which often target social relationships (Laird et al., 2018; Ryan et al., 2020). However, a lack of interventions focusing on leisure persists (Dequanter et al., 2021). This is surprising considering that over 65s spend approximately 7 hours a day on leisure activities (Payne, 2017), report a need for entertainment from technology (Egan & Pot, 2016), and report enjoyment using technology (Astell, 2013; Chirico et al., 2022; Lee et al., 2023). Leisure focused digital interventions do exist for people with dementia (Astell, 2019) but older adults tend to have negative attitudes towards gaming (Ferguson et al., 2016). Qualitatively, this review highlights evidence that pleasure can be derived from digital interventions, even when enjoyment is not their primary focus (Hicks et al., 2019; Rai, Prasetya, et al., 2021; Ryan et al., 2020) and therefore leisure-based technologies and making these more appealing to people with dementia requires further exploration.

This review also highlights the importance of creating personalised or tailored digital interventions for people with dementia so that they are desirable and accessible. General attitudes toward technology, which are underexplored, form part of this influence. Individuals with negative attitudes appear less motivated and less likely to engage and this is well documented as a barrier to digital intervention engagement (Christie et al., 2018; Pang et al., 2021; Smith & Mountain, 2012). Study recruitment for effectiveness testing is thus unavoidably biased, as individuals with positive attitudes, and motivation are more likely to take part in technology studies, unrepresentative of all people living with dementia. Echoing Holthe and colleagues (2018), future research should prioritise using participatory methods of intervention development, to better understand people’s attitudes towards technology and create interventions which are more desirable and accessible to all people with dementia. Also, given the importance of carer’s for supporting people with dementia to use digital interventions, like Bradley and colleagues (2023) we recommend that they too are considered when designing interventions.

## Conclusion

This review synthesised studies exploring effectiveness and experiences of people living with dementia or mild cognitive impairment using digital interventions to improve their quality of life. This shows people living with dementia can benefit from using digital interventions if they are motivated, and if delivery is tailored and supported. Delivery might be optimised by providing interventions at home using structured time and frequency instructions because this allows easier integration into lifestyles.

## Supplemental Material

Supplemental Material - Exploring the effectiveness and experiences of people living with dementia interacting with digital interventions: A mixed methods systematic review Supplemental Material for Exploring the effectiveness and experiences of people living with dementia interacting with digital interventions: A mixed methods systematic review by Annabel Ditton, Hissah Aloda, Christopher Fox, Shirley Evans, and Jane Cross in Dementia
