# Supplementary material for: Exploring the effectiveness and experiences of people living with dementia interacting with digital interventions: A mixed methods systematic review
Source: Dementia (London). 2024 Nov 27;24(3):506–51. doi: 10.1177/14713012241302371 (PMC11915779; doi:10.1177/14713012241302371)
Supplement: Supplemental Material - Exploring the effectiveness and experiences of people living with dementia interacting with digital interventions: A mixed methods systematic review [file sj-pdf-1-dem-10.1177_14713012241302371.pdf]

Exploring the effectiveness and experience of people with dementia interacting with digital interventions: A systematic review. – Search Strategy

Web of Science

((TS=(Dementia OR Alzheimer* OR "Mild cognitive impair*")) AND TS=("Digital Technology " OR "Mobile App*" OR "Video Games" OR Digital* OR Computer* OR "web based" OR "internet based" OR "online intervention" OR "virtual reality" OR "augument* reality")) AND TS=("Quality of Life" OR "Health related quality of life" OR Wellbeing OR Affect OR Mood OR Depress* OR "self esteem" Cognit* OR Memory OR "Executive function*" OR "family relation*" OR "social interact*" OR “social connect*” OR "Activities of Daily Living" OR Independe* OR “self efficacy” OR “self management” OR "self esteem" OR Exercise OR Fitness OR Mobil* OR "home environment")

MEDLINE

(MESH) Dementia/, ,

OR

(Dementia or Alzheimer* or "mild cogniti* impair*") ab,ti., ,

AND

(MESH) Digital Technology/, ,

(MESH) web-based Intervention/, ,

OR

("digital tech*" or "mobile app*" or "video gam*" or digital or computer* or "web based" or "internet based" or "online interventions" or "virtual reality" or "augument* reality").ab,ti., ,

AND

(MESH) Quality of Life"/, ,

OR

("quality of life" or "health related quality of life" or wellbeing or affect or mood or depress* or Cognit* or memory or "executive function*" or "family relation*" or "social interact*" or "Social connect*" or "Activit* of daily living" or independen* or "self efficacy" or "self management" or “self esteem” OR exercise or fitness or mobil* or "home environment").ab,ti., ,

EMBASE

(MESH) Dementia/, , ,

OR

(Dementia or Alzheimer* OR "mild cognitive impair*").ab,ti.

AND

(MESH) Digital Technology/, ,

(MESH) Internet-Based Intervention/, ,

OR

("Digital Technolog*" or "Mobile app*" or "video gam*" or digital* or computer* or "web based" or "internet based" or "online intervention" or "virtual reality" or "augument* reality").ab,ti.

AND

(MESH) Quality of Life"/, ,

OR

("quality of life" or "health related quality of life" or wellbeing or Affect or Mood or Depress* or cognit* or memory or "executive *function*" or "family relation*" or "social interact*" or "social connect*" or "activities of daily living" or independe* or "self efficacy" or "self management" or “self esteem” OR exercise or fitness or mobil* or "home environment").ab,ti.

PSYCHINFO

(MESH) Dementia/, , ,

OR

(Dementia or Alzheimer* OR "mild cognitve* impair*").ab,ti.

AND

(MESH) Digital Interventions”/, ,

OR

("Digital Technolog*" or "Mobile app*" or "video gam*" or digital* or computer* or "web based" or "internet based" or "online intervention" or "virtual reality" or "augument* reality").ab,ti.

AND

(MESH) Quality of Life"/, ,

OR

("quality of life" or "health related quality of life" or wellbeing or Affect or Mood or Depress* or cognit* or memory or "executive *function*" or "family relation*" or "social

interact*" or "social connect*" or "activities of daily living" or independe* or "self efficacy" or "self management" or “self esteem” OR exercise or fitness or mobil* or "home environment").ab,ti.

CINAHL

SU (dementia or alzheimer* or "mild cognitive impair*") AND SU ("Digital Technolog*" or "Mobile app*" or "video gam*" or digital* or computer* or "web based" or "internet based" or "online intervention" or "virtual reality" or "augument* reality") AND SU ("quality of life" or "health related quality of life" or wellbeing or Affect or Mood or Depress* or cognit* or memory or "executive *function*" or "family relation*" or "social interact*" or "social connect*" or "activities of daily living" or independe* or "self efficacy" or "self management" or "self esteem" or exercise or fitness or mobil* or "home environment")

JBI QUALITY ASSESSMENTS: QUALITATIVE STUDIES

Author (Year)	Q1	Q2	Q3	Q4	Q5	Q6	Q7	Q8	Q9	Q10	Total of ‘Yes’ in an article (%)	Comments
Berrett et al (2022)	N	Y	Y	Y	Y	N	N	Y	Y	Y	70	Does not indicate philosophical perspective.<br><br>No reference to researcher influence or cultural perspective.
Bielsten et al	N	Y	Y	Y	Y	Y	N	Y	Y	Y	80	Does not indicate philosophical perspective.

(2020)												No discussion on influence of the researcher
Critten & Kurcikova (2019)	Y	Y	Y	Y	Y	N	Y	Y	Y	Y	90	No reference to researcher cultural or theoretical stance
Flynn et al (2022)	Y	Y	Y	Y	Y	Y	Y	Y	Y	Y	100	
Hicks & Innes (2019)	Y	Y	Y	Y	Y	Y	Y	Y	Y	Y	100	
Øksnebjerg et al (2019)	N	CT	Y	N	N	N	N	N	Y	Y	30	No methodology/perspective established, appropriate qualitative methods (interviews) but analysis described as ‘general inductive approach’ no clear methodological approach used.<br><br>Not reliable method of collecting data for step 1 ‘note taking’.<br><br>Only direct participants in step 2 of study, no supporting evidence for steps 1, 3 and 4.<br><br>No reference to statement on theoretical or cultural perspective or how researcher influenced the research
Ryan et al (2020)	Y	Y	Y	Y	Y	N	Y	Y	Y	Y	90	No cultural or theoretical statement
Smith et al (2020)	Y	Y	Y	Y	Y	Y	Y	Y	Y	Y	100	
Beishon et al (2021)	Y	Y	Y	Y	Y	Y	Y	Y	Y	Y	100	

Total of 'Yes' per question (%)	66.6	88.8	100	88.8	88.8	55.5	66.6	88.8	100	100		

Y: yes; U: unclear; N: no

Q1: Is there congruity between the stated philosophical perspective and the research methodology?

Q2: Is there congruity between the research methodology and the research question or objectives?

Q3: Is there congruity between the research methodology and the methods used to collect data?

Q4: Is there congruity between the research methodology and the representation and analysis of data?

Q5: Is there congruity between the research methodology and the interpretation of results?

Q6: Is there a statement locating the researcher culturally or theoretically?

Q7: Is the influence of the researcher on the research, and vice-versa, addressed?

Q8: Are participants, and their voices, adequately represented?

Q9: Is the research ethical according to current criteria or, for recent studies, is there evidence of ethical approval by an appropriate body?

Q10: Do the conclusions drawn in the research report flow from the analysis, or interpretation, of the data?

JBI QUALITY ASSESSMENTS: MIXED METHODS STUDIES

**Author (Year)**	**Screening**		**Mixed method component**					**Qualitative component**					**Quantitative component**					**Total of 'yes' per article (%)**
	**S1**	**S2**	**Q5.1**	**Q5.2**	**Q5.3**	**Q5.4**	**Q5.5**	**Q1.1**	**Q1.2**	**Q1.3**	**Q1.4**	**Q1.5**	**Q2.1**	**Q2.2**	**Q2.3**	**Q2.4**	**Q2.5**	
Kerkhof et al (2022)	Y	Y	Y	Y	Y	Y	CT	Y	Y	Y	Y	CT	Y	Y	Y	Y	Y	88.2
													**Q3.1**	**Q3.2**	**Q3.3**	**Q3.4**	**Q3.5**	

Mattos et al (2021)	Y	Y	CT	Y	Y	Y	N	Y	Y	Y	N	Y	N	Y	Y	N	Y	70.5
König et al (2022)	Y	Y	Y	Y	Y	Y	N	Y	Y	CT	N	CT	Y	Y	CT	Y	Y	70.5
Berenbaum et al (2020)	Y	Y	Y	Y	Y	Y	Y	Y	Y	Y	Y	Y	Y	N	Y	CT	Y	88.2
Rai et al (2021a)	Y	Y	CT	N	Y	CT	Y	Y	Y	Y	Y	Y	Y	Y	CT	CT	Y	70.5
Rai et al (2021b)	Y	Y	Y	N	Y	Y	Y	Y	Y	Y	Y	Y	Y	Y	Y	Y	Y	88.2
Sheehy et al (2021)	Y	Y	Y	N	Y	CT	Y	Y	Y	Y	Y	Y	CT	CT	Y	Y	Y	76.4
Beentjes et al (2020a)	Y	Y	Y	Y	Y	Y	N	Y	Y	Y	CT	Y	Y	Y	Y	CT	Y	82.3
													**Q4.<br>1**	**Q4.<br>2**	**Q4.<br>3**	**Q4.4**	**Q4.5**	
Bogza et al (2020)	Y	Y	N	Y	Y	Y	CT	Y	Y	Y	Y	Y	Y	N	Y	N	Y	76.4
**Total of 'Yes' per question (%)**	100	100	66.6	66.6	100	77.7	44.4	100	100	88.8	66.6	77.7	77.7	66.6	77.7	44.4	100	

Y: yes; CT: can't tell; N: no

S1. Are there clear research questions?
S2. Do the collected data allow to address the research questions?
5.1. Is there an adequate rationale for using a mixed methods design to address the research question?
5.2. Are the different components of the study effectively integrated to answer the research question?
5.3. Are the outputs of the integration of qualitative and quantitative components adequately interpreted?
5.4. Are divergences and inconsistencies between quantitative and qualitative results adequately addressed?
5.5. Do the different components of the study adhere to the quality criteria of each tradition of the methods involved?

1.1. Is the qualitative approach appropriate to answer the research question?
1.2. Are the qualitative data collection methods adequate to address the research question?
1.3. Are the findings adequately derived from the data?
1.4. Is the interpretation of results sufficiently substantiated by data?
1.5. Is there coherence between qualitative data sources, collection, analysis and interpretation?
2.1 Is randomisation appropriately performed?
2.2 Are the groups similar at baseline?
2.3 are there complete outcome data?
2.4 Are the outcome assessor blind to the intervention provided?
2.5 Did the participants adhere to the assigned intervention?
3.1. Are the participants representative of the target population?
3.2. Are measurements appropriate regarding both the outcome and intervention (or exposure)?
3.3. Are there complete outcome data?
3.4. Are the confounders accounted for in the design and analysis?
3.5. During the study period, is the intervention administered (or exposure occurred) as intended?
4.1. Is the sampling strategy relevant to address the research question?
4.2. Is the sample representative of the target population?
4.3. Are the measurements appropriate?
4.4. Is the risk of nonresponse bias low?
4.5. Is the statistical analysis appropriate to answer the research question?

JBI QUALITY ASSESSMENTS: RANDOMISED CONTROL TRIALS															
**Author (Year)**	**Q1**	**Q2**	**Q3**	**Q4**	**Q5**	**Q6**	**Q7**	**Q8**	**Q9**	**Q10**	**Q11**	**Q12**	**Q13**	**Total of 'Yes' in an article (%)**	**Comments**
Yang et al (2022)	CT	CT	CT	N	CT	N	CT	Y	CT	Y	Y	Y	Y	38.5	Randomisation method not specified. Participant demographic means & SDs reported but no significance testing conducted. Control group had more sessions of intervention. Outcome measures standardised, but no report on how/who completed them.
Torpil et al (2021)	Y	CT	Y	N	N	Y	Y	Y	CT	Y	Y	CT	Y	61.5	No power analysis reported
Thapa et al (2020)	Y	CT	Y	N	N	N	CT	Y	Y	Y	Y	CT	Y	53.8	Slightly more males (n=4) in control, but not significant difference. Length of each session was different between control and intervention group. No power analysis
Singh et al (2022)	CT	CT	CT	CT	CT	CT	CT	CT	CT	Y	Y	N	Y	23	Randomisation method not specified. Says double blind but does not say how/who was blinded. Lack of reporting on procedure. P values below 0.05 not specified, does not report on the direction of effect. No power analysis
Shyu et al (2021)	Y	N	N	Y	N	N	N	Y	Y	Y	Y	Y	Y	61.5	Education, dementia severity, and depression differ but not sig on Mann-Whitney/Pearson's. Intervention group had more contact with healthcare team. Outcome assessor blind to 3 out of 4 measures. Pilot study, power analysis conducted but primary outcome not stated

Scullin et al (2021)	Y	CT	Y	N	N	CT	Y	Y	CT	Y	Y	Y	Y	61.5	Doesn't report who conducted the assessments. Location of at home use means unable to control other possible 'causes'
Schmitter-Edgecombe (2021)	N	CT	Y	CT	CT	Y	CT	Y	CT	Y	Y	Y	Y	53.8	Mostly random, but first 5 10 not random due to problem with intervention being ready.
Sautter et al (2021)	CT	CT	CT	CT	CT	CT	CT	Y	CT	Y	Y	N	Y	30.7	No report on blinding or randomisation for the relevant study just that randomised. Standardised measures but no statement about who completed measures. No power analysis to determine sample size
Rodella et al (2022)	Y	CT	Y	CT	CT	Y	Y	Y	Y	Y	Y	Y	Y	76.9	
Rai et al (2021c)	Y	Y	Y	N	N	CT	Y	Y	Y	Y	Y	Y	Y	76.9	Sample size used is consistent with previous pilot studies
Poptsi et al (2019)	Y	Y	Y	N	N	Y	Y	Y	CT	Y	Y	CT	Y	61.5	Standardised measures but no statement about who completed measures. No power analysis
Phatak et al (2021)	N	N	CT	N	CT	CT	Y	Y	CT	CT	Y	CT	Y	30.7	Subtractive randomisation method. Lack of detail on demographics to ascertain similarity of groups (only age & gender). Standardised measures but no statement about who completed measures. No description of attrition/reasons for attrition. No power analysis, participants were part of larger trial
Park et al (2020a)	CT	CT	Y	CT	CT	Y	Y	Y	CT	CT	Y	CT	Y	46.1	No power calculation, small sample size highlighted as a limitation by author, no mention of attrition rates
Park et al (2020b)	Y	CT	Y	N	N	CT	CT	Y	Y	Y	Y	CT	Y	53.8	Pilot study – no power analysis, low power highlighted as a limitation by author

Park, Jung & Lee (2020)	Y	Y	Y	N	N	CT	CT	Y	CT	Y	Y	Y	Y	61.5	No report on the training of person conducting assessment
Park (2022a)	Y	CT	Y	N	N	CT	Y	Y	Y	Y	Y	Y	Y	69.2	OT conducted assessment using valid tests.
Park (2022b)	Y	N	Y	N	N	Y	Y	Y	Y	Y	Y	Y	Y	76.9	
Nousia et al (2021)	CT	CT	Y	N	N	Y	Y	Y	Y	Y	Y	N	Y	61.5	Randomisation method not specified, not mention of concealment. Semantic fluency in control sig higher but rest of demographics similar. No power calculation – author reports possibly underpowered as limitation. Used T-test, ANOVA may be more appropriate.
Micarelli et al (2019)	Y	CT	CT	N	N	CT	Y	Y	Y	Y	Y	CT	Y	53.8	Did not measure at home adherence, unable to ascertain identical treatment & authors report different amounts of time spent in rehabilitation. No power calculation for sample size but small sample size considered during analysis
Marin et al (2022)	Y	N	Y	N	N	N	N	Y	Y	Y	CT	CT	Y	46.1	Does not report who conducted measures. High dropout led to lack of power at follow up for most measures so they could not be analysed – feasibility study, effectiveness was secondary aim
Manenti et al (2020)	Y	Y	N	N	CT	CT	Y	Y	Y	Y	Y	Y	Y	69.2	Gender significantly different between groups
Li et al (2019)	CT	CT	N	N	N	CT	Y	Y	Y	Y	CT	CT	Y	38.4	Gender and some subtest cognitive scores different between groups. No report on who conducted assessment. No power calculation but effect sizes reported
Lee et al (2018)	Y	Y	Y	Y	N	Y	Y	Y	Y	Y	Y	CT	Y	84.6	Pilot study so sample size based on previous studies but small.

Knoefel et al (2018)	CT	CT	CT	CT	CT	CT	CT	Y	CT	Y	Y	N	Y	30.78	Significance not tested for between baseline groups. Standardised tests used, but no report on who conducted assessments. Pilot not powered to detect change. Used multiple t-tests, ANOVA would be more appropriate.
Kim et al (2020)	Y	Y	Y	N	N	N	Y	Y	Y	Y	Y	CT	Y	69.2	Participants allowed to take medications as per usual care throughout. Pilot study not powered to establish efficacy just feasibility
Jirayucharoensak et al (2019)	CT	N	Y	N	N	CT	N	Y	Y	Y	CT	CT	Y	38.4	Does not report who conducted cog assessment. Power analysis not conducted.
Han et al (2020)	Y	N	Y	N	N	CT	N	Y	Y	Y	CT	CT	Y	38.4	Pilot study, no power calculation or justification for sample size. No report on who conducted assessments.
Flak et al (2019)	Y	Y	Y	Y	CT	Y	Y	Y	Y	Y	CT	Y	Y	84.6	No reference to blinding of those delivering treatment or who conducted outcome assessments
Duff et al (2021)	Y	Y	Y	Y	Y	Y	Y	Y	Y	Y	CT	CT	Y	84.6	More females in treatment group but not significantly different. Control group used more hours than intervention group. Mood scale not used standardised scale; rest have but not report on who gathered data. Authors conducted power analysis but did not recruit enough p's but still had 80% power.
Cheng et al (2022)	Y	Y	Y	Y	N	Y	Y	Y	Y	Y	Y	CT	Y	84.6	Small sample size with no power calculation
Cavallo and Angilletta (2019)	Y	CT	CT	N	N	Y	Y	Y	Y	CT	Y	CT	Y	53.8	No demographic data provided, prev. study was except 2 cognition scores, however as 4 ppts dropped out before the follow ups not sure how this influences sample used. No follow up data

															on HADS. No power calculation.
Baquero et al (2022)	Y	CT	Y	N	N	CT	Y	Y	Y	Y	CT	CT	Y	53.8	No power calculation. No report of how/who conducted assessments
Amjad et al (2019)	CT	CT	CT	CT	CT	Y	Y	Y	CT	Y	Y	CT	Y	46.1	No information on blinding or concealment. No power calculation. No report on who conducted assessment
Beentjes et al (2020b)	CT	N	Y	N	N	N	N	Y	Y	Y	Y	CT	Y	46.1	Pilot feasibility study - did not recruit enough p's based on power calculation
Total of 'Yes' per question (%)	65.7	25.7	68.5	14.2	2.8	42.8	62.8	97.1	62.8	91.4	80	34.2	100		

Y: yes; CT: can't tell; N: no

Q1. Was true randomization used for assignment of participants to treatment groups?
Q2. Was allocation to treatment groups concealed?
Q3. Were treatment groups similar at the baseline?
Q4. Were participants blind to treatment assignment?
Q5. Were those delivering the treatment blind to treatment assignment?
Q6. Were treatment groups treated identically other than the intervention of interest?
Q7. Were outcome assessors blind to treatment assignment?
Q8. Were outcomes measured in the same way for treatment groups?
Q9. Were outcomes measured in a reliable way
Q10. Was follow up complete and if not, were differences between groups in terms of their follow up adequately described and analysed?
Q11. Were participants analysed in the groups to which they were randomized?
Q12. Was appropriate statistical analysis used?
Q13. Was the trial design appropriate and any deviations from the standard RCT design (individual randomization, parallel groups) accounted for in the conduct and analysis of the trial?

JBI QUALITY ASSESSMENTS: NON-RANDOMISED CONTROL TRIALS											
**Author (Year)**	**Q1**	**Q2**	**Q3**	**Q4**	**Q5**	**Q6**	**Q7**	**Q8**	**Q9**	**Total of 'Yes' in an article (%)**	**Comments**
Zhu et al (2022)	Y	Y	Y	N	Y	Y	Y	CT	CT	66.6	MCI vs MD, all similar except cognition but analysed within groups. Multiple cog measures, reliable measures but no info on who conducted & used same versions of test so possible learning effect. No power analysis
Zhang et al (2019)	Y	Y	Y	N	Y	Y	Y	CT	CT	66.6	No report on how conducted the analysis. No effect sizes or power analysis
Zajac-Lampaska et al (2019)	Y	Y	Y	N	Y	Y	Y	CT	N	77.7	Healthy vs dementia but within group analysis. Doesn't reference how and who completed the testing but did use standardised tests. Effect sizes reported but no power analysis to determine needed sample size – power was low in mild dementia group
Van Stanten et al (2020)	Y	Y	Y	Y	Y	Y	Y	Y	Y	100	Powered on the secondary outcome (MMSE)
Tsolaki et al (2020)	Y	Y	CT	Y	Y	Y	Y	CT	CT	66.6	No power calculation, clear explanation of data treatment.
Taylor et al (2019)	Y	Y	Y	N	Y	Y	Y	Y	CT	77.7	Pilot feasibility study, no reference to whether pilot is powered to detect changes
Petersen et al (2020)	Y	Y	CT	Y	N	Y	Y	CT	CT	55.5	Can't control how intervention was used when in home environment. Doesn't reference how and who completed the testing but did use standardised tests. Pilot study but no power analysis but primary outcome is efficacy

Maeng et al (2021)	Y	Y	N	N	N	Y	Y	Y	CT	55.5	MCI vs Healthy so sig difference but within group analysis conducted. No power analysis
Laird et al (2018)	Y	Y	Y	N	Y	Y	Y	Y	Y	88.8	Feasibility pilot to determine power needed for RCT. Reports that recruitment numbers were in line with recommendations for feasibility trials
Kim et al (2021)	Y	Y	Y	N	N	Y	Y	CT	CT	55.5	MCI vs healthy, similar other than cognition and within group analysis. Doesn't reference how and who completed the testing but did use standardised tests. Did not conduct power analysis, no effect sizes.
Ferry et al (2020)	Y	Y	Y	N	Y	Y	Y	Y	CT	77.7	Pilot cost effectiveness study. No power analysis. Lack of reporting on what analysis was completed
Debring et al (2021)	Y	Y	Y	N	Y	Y	Y	CT	CT	66.6	Doesn't reference how and who completed the testing but did use standardised tests.<br><br>No power analysis completed no sensitivity analysis
Cinar & Sahinar (2020)	Y	Y	CT	Y	Y	Y	Y	CT	Y	77.7	Individuals may have used intervention from different times (3 months or 1200mins).<br><br>Doesn't reference how and who completed the testing but did use standardised tests.<br><br>No power analysis<br><br>Drop out reasons not reported
Burgos-Morelos et al (2023)	Y	N	Y	N	N	Y	Y	CT	CT	44.4	Doesn't reference how and who completed the testing but did use standardised tests. No power analysis. Not all significant differences were mentioned in results
Bojan et al (2021)	Y	Y	Y	N	Y	Y	Y	CT	N	66.6	No sample size calc – used rule of thumb (n=10-30) due to being a pilot. ANOVA might have been more

											appropriate
Chen et al (2022)	Y	Y	CT	N	N	Y	Y	CT	CT	44.4	Calculation of sample size referenced but lack of detail. No effect sizes. No report on how confounding variables may impact
Total of ‘Yes’ per question (%)	100	93.7	68.7	25	11	68.7	100	14.2	8.5		

Y, Yes; N, No; CT, Can't tell

Q1. Is it clear in the study what is the ‘cause’ and what is the ‘effect’ (i.e. there is no confusion about which variable comes first)?

Q2. Were the participants included in any comparisons similar?

Q3. Were the participants included in any comparisons receiving similar treatment/care, other than the exposure or intervention of interest?

Q4. Was there a control group?

Q5. Were there multiple measurements of the outcome both pre and post the intervention/exposure?

Q6. Was follow up complete and if not, were differences between groups in terms of their follow up adequately described and analyzed?

Q7. Were the outcomes of participants included in any comparisons measured in the same way?

Q8. Were outcomes measured in a reliable way?

Q9. Was appropriate statistical analysis used?

JBI QUALITY ASSESSMENTS: CASE STUDY DESIGN										
**Author (Year)**	**Q1**	**Q2**	**Q3**	**Q4**	**Q5**	**Q6**	**Q7**	**Q8**	**Total of 'Yes' in an article (%)**	**Comments**
Imbeault et al (2018a)	Y	Y	Y	Y	Y	Y	Y	CT	87.5	
Imbeault et al (2018b)	Y	Y	Y	Y	Y	Y	Y	CT	87.5	
Foloppe et al (2018)	Y	Y	Y	Y	Y	Y	Y	Y	100	
Edgar & Bargmann (2021)	Y	Y	Y	Y	Y	N	Y	Y	87.5	
Total of 'Yes' per question (%)	100	100	100	100	100	75	100	50		

Y, Yes; N, No; CT, Can't tell

Q1. Were patient's demographic characteristics clearly described?

Q2 Was the patient's history clearly described and presented as a timeline?

Q3 Was the current clinical condition of the patient on presentation clearly described?

Q4 Were diagnostic tests or assessment methods and the results clearly described?

Q5 Was the intervention(s) or treatment procedure(s) clearly described?

Q6 Was the post-intervention clinical condition clearly described?

Q7 Were adverse events (harms) or unanticipated events identified and described?

Q8 Does the case report provide takeaway lessons?

JBI QUALITY ASSESSMENTS: CASE SERIES DESIGN												
**Author (Year)**	**Q1**	**Q2**	**Q3**	**Q4**	**Q5**	**Q6**	**Q7**	**Q8**	**Q9**	**Q10**	**Total of 'Yes' in an article (%)**	**Comments**
Fasilis et al (2018)	Y	Y	Y	N	N	N	N	Y	CT	Y	50	Inclusion criteria vaguely reported. Setting only named, no further explanation as to what the setting is

Y, Yes; N, No; CT, Can't tell

Q1. Were there clear criteria for inclusion in the case series?

Q2. Was the condition measured in a standard, reliable way for all participants included in the case series?

Q3. Were valid methods used for identification of the condition for all participants included in the case series?

Q4. Did the case series have consecutive inclusion of participants?

Q5. Did the case series have complete inclusion of participants?

Q6. Was there clear reporting of the demographics of the participants in the study?

Q7. Was there clear reporting of clinical information of the participants?

Q8. Were the outcomes or follow up results of cases clearly reported?

Q9. Was there clear reporting of the presenting site(s)/clinic(s) demographic information?

Q10. Was statistical analysis appropriate?
